# Intestinal *Lactobacillus johnsonii* protects against neuroangiostrongyliasis in BALB/c mice through modulation of immune response

**DOI:** 10.1371/journal.pntd.0012977

**Published:** 2025-04-08

**Authors:** Long Yin Lam, Ting-Ruei Liang, Wen-Jui Wu, Ho Yin Pekkle Lam

**Affiliations:** 1 State Key Laboratory of Chemical Biology and Drug Discovery, Department of Applied Biology and Chemical Technology, The Hong Kong Polytechnic University, Kowloon, Hong Kong SAR, China; 2 PhD Program in Pharmacology and Toxicology, Tzu Chi University, Hualien, Taiwan; 3 Department of Laboratory Medicine and Biotechnology, Tzu Chi University, Hualien, Taiwan; 4 Department of Biochemistry, School of Medicine, Tzu Chi University, Hualien, Taiwan; 5 Institute of Medical Sciences, Tzu Chi University, Hualien, Taiwan; Zhejiang Wanli University, CHINA

## Abstract

Neuroangiostrongyliasis is characterized by eosinophilic meningoencephalitis with a robust onset of severe neurological symptoms, by which immunological factors and peripheral metabolites have been postulated to affect the course of the disease. The gut-brain axis provides a bidirectional communication between the gut and the central nervous system, and therefore, understanding the gut microbiome may provide us with a deeper insight into the pathogenesis of angiostrongyliasis. Using 16S rRNA sequencing, we identified an increase in the abundance of different *Lactobacillus* species in *Angiostrongylus cantonensis*-infected mice, which was correlated to the disease severity. However, attempts to inoculate *L. johnsonii* into *A. cantonensis*-infected mice surprisingly revealed an improvement in neuroinflammation and prolonged survival. RNA sequencing suggested an immune-modulatory effect of *L. johnsonii*, which was confirmed by ELISA, showing increased levels of IL-10 and reduced levels of IL-2, IL-4, IL-5, and MCP-1 in the brain. Nevertheless, *L. johnsonii*-associated improvements were not associated with microbiome-related metabolites, as UHPLC-MS/MS analysis revealed no change in short-chain fatty acids, tryptophan metabolites, and bile acids. Our results suggest that while intestinal *L. johnsonii* appears to be linked to the progression of neuroangiostrongyliasis, these bacteria are likely attempting to modulate the dysregulated immune response to combat the disease. This is one of the first studies to investigate the gut microbiome in mice with *A. cantonensis* infection, which extends our knowledge from the microbiome-point-of-view of the pathogenesis of angiostrongyliasis and how the body defends against *A. cantonensis*. This work also extends to possible treatment approaches using *L. johnsonii* as probiotics.

## Author summary

The gut-brain axis provides a bidirectional communication between the gut and the central nervous system and, therefore, is related to multiple disease pathogenesis. This research aimed to analyze the microbiome change of mice with *Angiostrongylus cantonensis* infection. Our results suggested an increase in the abundance of *Lactobacillus* in *A. cantonensis*-infected mice, which was initially thought to be correlated with the disease severity. However, oral supplementation of *Lactobacillus johnsonii* surprisingly improved the disease and the survival of *A. cantonensis*-infected mice. Further analysis suggested that gavage of *L. johnsonii* increased IL-10 and decreased IL-2, IL-4, IL-5, and MCP-1 levels in the brain. Our results provide evidence of how the gut microbiome affects angiostrongyliasis and support that *Lactobacillus* may represent a potential probiotic that may improve angiostrongyliasis and may serve as a new treatment approach.

## Introduction

Neuroangiostrongyliasis is characterized by eosinophilic meningoencephalitis with a robust onset of severe neurological symptoms. The disease has a high mortality rate, and even if patients survive, long-term neurological sequelae usually occur [1]. The lack of specific drugs for neuroangiostrongyliasis, with only a combination of albendazole and steroids showing limited efficacy [2], underscores the urgency and importance of further research. Immunological factors have been postulated to affect the course of the disease [1,2], including cytokines such as IL-33 and IL-5 that orchestrate the occurrence of eosinophilic inflammation [3]. In addition, peripheral metabolites have also been shown to affect neurological diseases, by which they permeate the blood-brain barrier and modulate transcriptional and epigenetic programs of brain cells [4].

The gut microbiome has been known as a major contributor to immune modulation and the production of metabolites. It has been known to positively or negatively affect neurological diseases, where they may directly produce specific metabolites or indirectly affect the secretion of cytokines or chemokines from the immune cells [5]. The gut-brain axis provides a bidirectional communication between the gut and the central nervous system (CNS) [5], and therefore, understanding the modulatory effect of the gut microbiome may provide us with a deeper insight into the pathogenesis of neurological diseases. To our knowledge, there has been no investigation into how the gut microbiome affects the course of neuroangiostrongyliasis.

Of note, *Lactobacillus* is one of the major bacteria commonly found in human gut microbiome [6]. It is also widely used in food production for its ability to produce lactic acid and has long been recognized as a probiotic due to its functional benefits, including enhancement of nutritional absorption, modulation of inflammation and immunity, and defense against pathogens [7]. This bacteria has been suggested to benefit diseases such as inflammatory bowel disease [8], neuroinflammation [9], and arthritis [10]. Although *Lactobacillus* shows promise in human health and disease prevention, it is also associated with adverse effects. In immunocompromised patients, *Lactobacillus* has been implicated as a pathogen capable of causing infective endocarditis, bacteremia, or local infections [11]. Therefore, further knowledge of this bacterium and its role in different diseases is necessary. In this study, we provided preliminary animal data to assess the potential involvement of the *Lactobacillus* in neuroangiostrongyliasis.

## Materials and methods

### Ethics statement

All experimental protocols involving animals were reviewed and approved by the Institutional Animal Care and Use Committees (IACUC) of Tzu Chi University (No. 104076-A and 112007). All animal experiments were carried out under approved guidelines of the National Institutes of Health (NIH) Guide for the Care and Use of Laboratory Animals (ISBN 0-309-05377-3, 1996).

### Animal and *Angiostrongylus cantonensis*

Male BALB/c mice and Sprague-Dawley rats were obtained from the National Laboratory Animal Center, Taipei, Taiwan and were housed in the animal facility at Tzu Chi University under standardized husbandry conditions.

The life cycle of *Angiostrongylus cantonensis* was maintained under laboratory conditions as previously described [12]. Sprague-Dawley rats were used as the final host and the freshwater snail *Biomphalaria glabrata* was used as the intermediate host. To infect BALB/c mice for experiments, infected *B. glabrata* were digested by 0.6% acid pepsin (pH 2) to release third-stage larvae (L3). The L3 was counted into a glass well, mixed with sterile water, and introduced to the mice by oral gavage. After each gavage, the glass well was checked for any remaining L3.

### Bacterial cultures

*Lactobacillus johnsonii* (ATCC #33200) was grown in the MRS broth supplemented with 0.05% cysteine under anaerobic conditions (75% N_2_, 20% CO_2_, 5% H_2_) at 37°C. *Lactococcus lactis* was isolated from a commercial probiotic product from Jamieson and was grown in the blood agar plate under aerobic conditions at 37°C. The bacteria were validated for purity by 16S Sanger sequencing (S1 Fig and S1 Data).

### Animal experiments

Male BALB/c mice were six weeks old at the beginning of all experiments. The sample size was predertimened by the resource equation [13]. To assess the role of the gut microbiome in mice with angiostrongyliasis, twelve mice were divided into two groups – the uninfected group and the infected group. Mice were infected with 25 L3 orally, whereas the uninfected control group was fed the same amount of sterile water. All the mice were sacrificed at week four post-infection. Upon sacrifice, blood, stool, and brain tissue were collected. Mice were singly housed in identical cages before stool collection. The brain was divided into two hemispheres for histology and biochemical analysis.

To investigate the potential role of *L. johnsonii* (four or six mice per group) or *L. lactis* (three mice per group) in angiostrongyliasis, BALB/c mice were infected with 25 L3. Starting two weeks post-infection, the mice were orally administered 100 μL of PBS-suspended *L. johnsonii* (OD = 1) or *L. lactis* (OD = 1) daily for two weeks. The same volume of sterile water was administered as the vehicle to the control mice. All the mice were sacrificed at week four post-infection.

### Neurofunctional tests

Mice were subjected to neurofunctional tests at the beginning of the experiment and every seven days. Neurological functions were evaluated by a modified version of neurological severity scores. Motor ability was assessed by observing the movement after hanging the mice and their walking posture. Motor coordination and balance were evaluated by the beam walking test. The mice were also tested for pinna reflex, corneal reflex, and the presence of seizures, myoclonus, and myodystony. A detailed scoring system is shown in S1 Table.

### Histological processing and examination

Brains were fixed in 10% neutral buffered formalin and dehydrated in a series of graded dilutions of alcohols. The tissues were cut into thin sections after immersion in xylene and molten paraffin. Slides were stained with hematoxylin and eosin (H&E) as described previously [14]. H&E-stained sections were examined and scored for the following parameters: meningitis, hemorrhage, and encephalitis. Each criterion was scored as 0, absent; 1, mild; 2, moderate; and 3, pronounced. At least ten random fields were examined in each section.

### Serology analysis

Brain tissues were homogenized on ice and centrifuged at 1500 × g at 4°C for 15 min to collect the supernatant. The protein concentrations of brain homogenate were determined by the Bradford method using a Bio-Rad Protein Assay Dye (Bio-Rad Laboratories, Hercules, CA, USA). Concentrations of interleukin (IL)-1β (Cat#: 432604; BioLegend, San Diego, CA, USA), IL-2 (Cat#: 431004; BioLegend), IL-4 (Cat#: 431104; BioLegend), IL-5 (Cat#: 431204; BioLegend), IL-10 (Cat#: 431414; BioLegend), monocyte chemoattractant protein-1 (MCP-1; Cat#: 88–7391; Thermo Fisher Scientific, Waltham, Massachusetts, USA), and gamma-aminobutyric acid (GABA; Cat#: EM1603; FineTest, Wuhan Fine Biotech, China) in sera and brain homogenates were measured using a standard sandwich ELISA kit.

### DNA extraction, 16S rRNA sequencing, and analysis

Total DNA from stool samples was extracted with a QIAamp DNA Stool Mini Kit (QIAGEN, Germantown, MD, USA) following the manufacturer’s instructions. DNA purity and concentration were monitored by a Qubit dsDNA HS assay kit (Invitrogen, Carlsbad, CA, USA). PCR amplification was performed spanning the V3 and V4 regions using the forward primers 5’-CCTACGGRRBGCASCAGKVRVGAAT-3’ and reverse primers 5’-GGACTACNVGGGTWTCTAATCC-3’ of the 16S rRNA gene and subsequently sequenced using 250 bp paired-end sequencing (Illumina MiSeq, Illumina, San Diego, USA).

Bcl2fastq software version 2.20 was used to analyze the original image data. Overlapping paired-end FASTQ files were matched and processed in QIIME version 1.9.1. Adapter sequences were removed by Cutadapt version 1.9.1 before the resulting sequence data were aligned to the database. Operational taxonomic unit (OTU) clustering was done by QIIME version 1.9.1 and VSEARCH version 1.9.6. The Ribosomal Database Project (RDP) classifier, Bayesian algorithm, was used to classify the OTU representative sequences with a threshold set at a 97% similarity level. Taxonomic annotation was assigned based on the Silva_138 16S rRNA database (http://www.arbsilva.de/), ITS database (https://unite.ut.ee/), and NCBI database (https://www.ncbi.nlm.nih.gov/). Alpha diversity and beta diversity were calculated by QIIME version 1.9.1. Ordination plots were calculated from the Brary-Curtis distance matrix using principal coordinate analysis (PCoA). LDA Effect Size (LEfse) version 1.0 and STAMP version 2.1.3 were used to analyze differentially different microbiomes between groups.

All the sequencing data were deposited in the Sequence Read Archive (SRA) database under the BioProject ID: PRJNA1169206 and BioSample accession: SAMN44067526–37.

### Real-time quantitative PCR (RT-qPCR)

Real-time quantitative PCR (RT-qPCR) was performed using a 2× qPCRBIO SyGreen Blue Mix Lo-ROX kit (PCR Biosystems, London, UK). PCR conditions for bacterial genes were 95°C for 2 min, followed by 50 cycles of 95°C for 10 s, 55°C for 10 s, and 72°C for 30 s. PCR conditions for mouse genes were 95°C for 2 min, followed by 50 cycles of 95°C for 10 s, 60°C for 15 s, and 72°C for 25 s. Data were analyzed using the 2^-(∆∆Ct)^ method with 16S (for bacterial genes) or GAPDH (for mouse genes) as the housekeeping gene. The primer pairs used are shown in the S2 Table.

### RNA extraction and sequencing

Total RNA was extracted from the brain tissue using TRIzol reagent (Invitrogen, Thermo Fisher Scientific) per the manufacturer’s protocol. One microgram of total RNA was used for the library preparation using oligo(dT) capture beads (Illumina) according to the manufacturer’s instructions. The double-stranded cDNA was treated for ends repair, dA-tailing, and adaptor ligation. Size selection of adaptor-ligated DNA was performed using DNA clean beads. Each sample was amplified by PCR using primers P5: 5’ AAT GAT ACG GCG ACC ACC GA 3’ and P7: 5’ CAA GCA GAA GAC GGC ATA CGA GAT 3’. Libraries with different indexes were multiplexed and sequenced using a 2×150 paired-end sequencing (Illumina).

FASTQ files were quality-filtered using Cutadapt version 1.9.1 and FastQC version 0.10.1. Alignment was done by Hisat2 version 2.2.1. Gene expression analysis and differential expression analysis were done by HTSeq version 0.6.1 and DESeq2 version 1.6.3, respectively. Adjusted *p*-values were set less than or equal to 0.05 to detect differentially expressed genes. GOSeq version 1.34.1 was used to identify Gene Ontology (GO) terms of enriched genes with adjusted *p*-values less than or equal to 0.05. Pathway enrichment analysis was performed using the Kyoto Encyclopedia of Genes and Genomes (KEGG) in-house scripts (https://www.genome.jp/kegg/pathway.html). Gene set enrichment analysis (GSEA) was performed based on sorting of GO terms using GSEA version 4.3.3.

All the sequencing data were deposited in the Sequence Read Archive (SRA) database under the BioProject ID PRJNA1169206 and BioSample accession SAMN44079053–54.

### Metabolomics analysis by ultra-high-performance liquid chromatography-tandem mass spectrometry (UHPLC-MS/MS)

The serum sample was mixed with methanol for protein precipitation. Subsequently, the sample was centrifuged at 18,000 × g for 10 min at 4°C. Sixty microliters of the supernatant were collected and mixed with 30 μL of 0.2 M 3- nitrophenylhydrazine (3-NPH) and 30 μL of 0.12 M N-(3-dimethylaminopropyl)-N’-ethylcarbodiimide hydrochloride (EDC) for derivatization. The mixture was allowed to react at 40°C for 20 min. Afterward, the sample was cooled on ice, followed by adding 30 μL of ^13^C-labeled 6–3-Nitrophenylhydrazine (^13^C6-3NPH) internal standard. Finally, the sample was filtered through a 0.22 μm cellulose membrane (Sartorius Stedim, Taipei, Taiwan) and analyzed by UHPLC-MS/MS.

UHPLC was performed by Agilent 1290 Infinity II UHPLC (Agilent Technologies, Santa Clara, CA). Metabolites were separated on an Agilent ZORBAX Eclipse Plus C18 column (2.1×100 mm; 1.8 μm particle size; Agilent Technologies) at 55°C using a 20-minute program. The mobile phase consisted of water (A) and isopropanol alcohol/acetonitrile (at a ratio of 3:1; B), each containing 0.1% formic acid. Gradient conditions were: 0-1.7 min, 0% B; 1.7-9.0 min, 15% B; 9.0-9.1 min, 30% B; 9.1-16 min, 30% B; 9.1-16.0 min, 40% B; 16.0-16.1 min, 72% B; 16.1-16.2 min, 80% B; 16.2-18.3 min, 100% B; 18.3-20.0 min, 0% B. Injection volume was 5 μL and flow rate was 0.35 mL/min.

MS/MS analysis was performed on an Agilent 6495C triple quadrupole mass spectrometer (Agilent Technologies). Reaction monitoring was conducted in the negative-ion mode. Setting parameters included: desolvation temperature, 200°C; desolvation gas flow, 11 l/min; sheath gas temperature, 250°C; sheath gas flow, 11 l/min; nebulizer pressure, 20 psi; capillary voltage, 3000 V; nozzle voltage, 1500 V; high-pressure radio frequency (RF) powers, 150 V; low-pressure RF powers, 60 V; multiplier voltage (Delta EMV), 750 V. Calibration curves were assessed with 6–8 levels of standards covering different linear range. The calibration equations were generated using a quadratic fit with a weighting factor of 1/x including the origin (except for lithocholic acid, which is 1/x^2). The correlation coefficients (R^2^) for all target analytes were greater than 0.995.

### Statistical analysis

Data are expressed as mean ± S.D unless stated otherwise. An unpaired t-test or Welch’s t-test was used to compare the differences in bacterial strain abundance between the two samples. One-way ANOVA, followed by Tukey’s honest significant difference (HSD) test, was used to compare between different groups. *p* < 0.05 were considered significant. PCoA analysis was performed and plotted based on the Brary-Curtis distance matrix. Pearson correlation analysis was calculated to evaluate the linear relationship between two variables. The probability of survival was calculated using the Kaplan–Meier method, with a log-rank test to analyze the differences between survival curves. All analyses were done by R software version 3.3.1, STAMP version 2.1.3, or GraphPad Prism version 9.4.1.

## Results

### *Angiostrongylus cantonensis* infection induces meningoencephalitis in BALB/c mice

**Fig 1:**
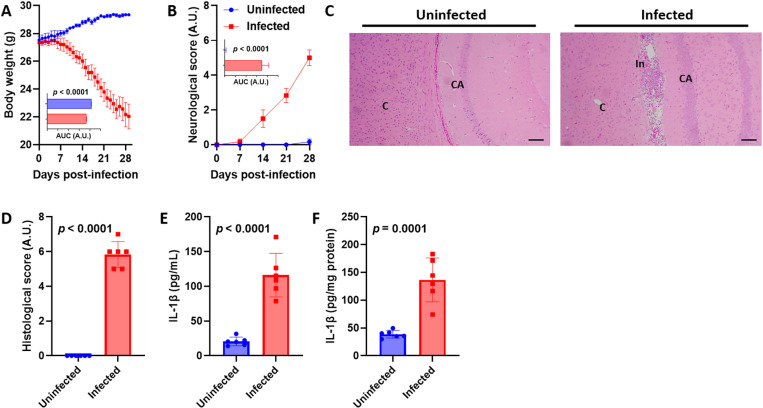
*Angiostrongylus cantonensis* infection induces brain inflammation. (A) The body weight of mice. (B) Neurological score of mice. (C) Representative H&E-stained section of the mouse brain. C, cerebral cortex; CA, cornu ammonis; In, inflammatory infiltration. Scale bars correspond to 200 μm. (D) Histological score of the brain sections. (E-F) Serum (E) and brain (F) interleukin (IL)-1β levels. ****n**** = 6 mice in each group. Data in (A and B) are presented as mean ± S.E.M and (D-F) as mean ± S.D; area under curve (AUC) data are presented as mean ± S.D. A.U., arbitrary unit. Significance determined by unpaired T-test, with ***p*** < 0.05 considered significant.

To assess the role of the gut microbiome during angiostrongyliasis, BALB/c mice were infected with 25 *A. cantonensis* L3. The body weight of infected mice was significantly decreased compared to that of the uninfected mice (Fig 1A). Neurological functions assessed by different neurological tests (S1 Table) suggested a time-dependent worsening in *A. cantonensis*-infected mice, with a progressively larger impairment of motor functions and balance as the disease progresses (Fig 1B). In agreement with this finding, histological analysis revealed significant meningoencephalitis (Fig 1C and 1D). Notably, IL-1β measured in the serum (Fig 1E) and brain homogenates (Fig 1F) also suggested a significant inflammatory response in the infected mice.

### *Angiostrongylus cantonensis* infection alters taxonomic composition in gut microbiome communities in BALB/c mice

**Fig 2:**
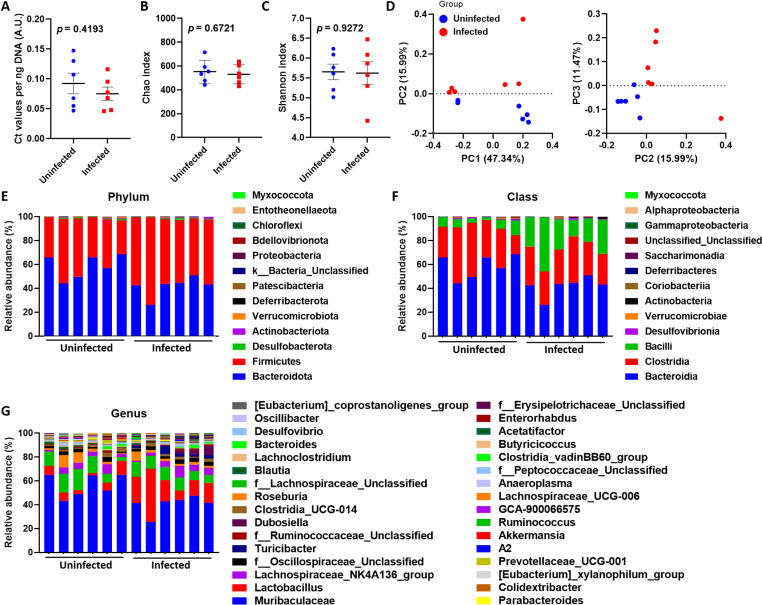
*Angiostrongylus cantonensis* infection induces changes in the gut microbiome. (A-C) Alpha diversity of the microbiomes in uninfected and infected mice. (A) qPCR-based quantification of total 16S copy-number in 1 ng of stool DNA, as shown by Ct values per ng DNA. (B) Chao index, for estimation of the number of OTUs per sample. (C) Shannon index, for estimation of the microbial diversity in each sample. (D) Principal co-ordinates analysis (PCoA) based on Bray-Curtis distance on the mice’s microbiomes between uninfected and infected mice. The x and y axes represent the first, second, and third principal coordinates. The value in percentage in the axis label represents the contribution of the corresponding coordinate to the sample variance. (E-G) Taxa summary of bacterial phyla (E), class (F), and genera (G; showing only the top 32 most abundant classifications) from uninfected and infected mice. ****n**** = 6 mice in each group. Data in (A-C) are presented as mean ± S.D. A.U., arbitrary unit. Significance determined by unpaired T-test, with ****p**** < 0.05 considered significant.

The suggested gut–microbiota–brain axis prompted us to investigate the locally prevalent microbiome community that potentially modulates the course of angiostrongyliasis. Therefore, 16S rDNA sequencing was used to assess the fecal microbiome composition between uninfected mice and *A. cantonensis*-infected mice. The total fecal bacterial load did not vary between uninfected and infected mice (Fig 2A). The same can be seen in the richness (Fig 2B) and diversity (Fig 2C) in stool samples between uninfected and infected mice, suggesting the overall microbial communities within each group were similar. However, principal co-ordinates analysis (PCoA) indicated that the gut microbiome configurations and composition between uninfected and infected mice were different (Fig 2D).

Analysis at the bacterial phyla level revealed significant changes in *Bacteroidota* (*p*-value = 0.010) and *Firmicutes* (*p*-value = 0.011; Fig 2E and S3 and S4 Tables) between uninfected and infected mice. At a class level, significant changes in *Bacteroidia* (*p*-value = 0.010) and *Bacilli* (*p*-value = 0.002) were observed between the two groups (Fig 2F and S3 and S4 Tables). When analyzing the bacterial genus between the two groups, changes were observed in *Muribaculaceae*, which is lower in the infected mice (*p*-value = 0.010), and *Lactobacillus*, which is higher in infected mice (*p*-value = 0.0258; Fig 2G and S3 and S4 Tables). These results were confirmed by LEfSe analysis (*p*-value = 0.010 for *Muribaculaceae* and *p*-value = 0.006 for *Lactobacillus*; S2A Fig and S5 Table) and STAMP analysis (*p*-value = 0.004 for *Muribaculaceae* and *p*-value = 0.042 for *Lactobacillus*; S2B Fig and S5 Table). These results suggest that an imbalance of the gut microbiome may modulate the disease progression of angiostrongyliasis.

### *Lactobacillus* species correlates to the progression of angiostrongyliasis

**Fig 3:**
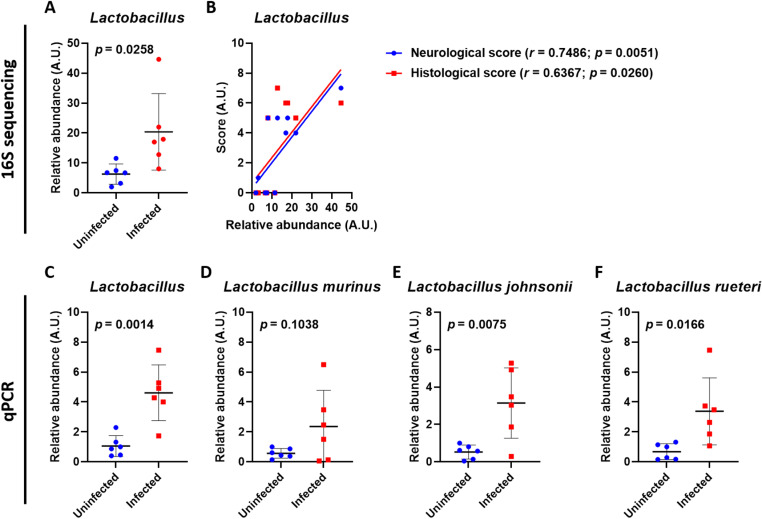
*Angiostrongylus cantonensis* infection alters intestinal *Lactobacillus* abundance. (A) Relative abundance of *Lactobacillus* between uninfected and infected mice, obtained by 16S rDNA sequencing of stool samples. (B) Scatter plot of Pearson’s correlation coefficient (*r*) between *Lactobacillus* abundance and neurological or histological scores. (C-F) Relative abundance of *Lactobacillus* (C), *Lactobacillus murinus* (D), *Lactobacillus johnsonii* (E), and *Lactobacillus reuteri* (F) between uninfected and infected mice, obtained by qPCR of stool samples. ****n**** = 6 mice in each group. Data in (A, C-F) are presented as mean ± S.D. A.U., arbitrary unit. Significance determined by unpaired T-test (A, C-F) or Pearson correlation (B), with ****p**** < 0.05 considered significant.

While sequencing results of the *Lactobacillus* (Figs 3A and S2) showed a significant positive correlation to neurological and histological scores of the mice (Fig 3B), validation of the abundance was done by qPCR (Fig 3C), which showed similar results. Further investigation at a species level showed that all identified *Lactobacillus* species had higher abundance during *A. cantonensis* infection (S3 Table), which was validated by the qPCR (Fig 3D-F). Pearson correlation analysis was performed to determine whether these lactobacilli may be related to disease features. The results revealed a strong positive correlation between different *Lactobacillus* species and neurological symptoms and brain histopathology (S3A-C Fig), but not with brain inflammation (S3D Fig).

### *Lactobacillus johnsonii* colonization improves angiostrongyliasis and survival

**Fig 4:**
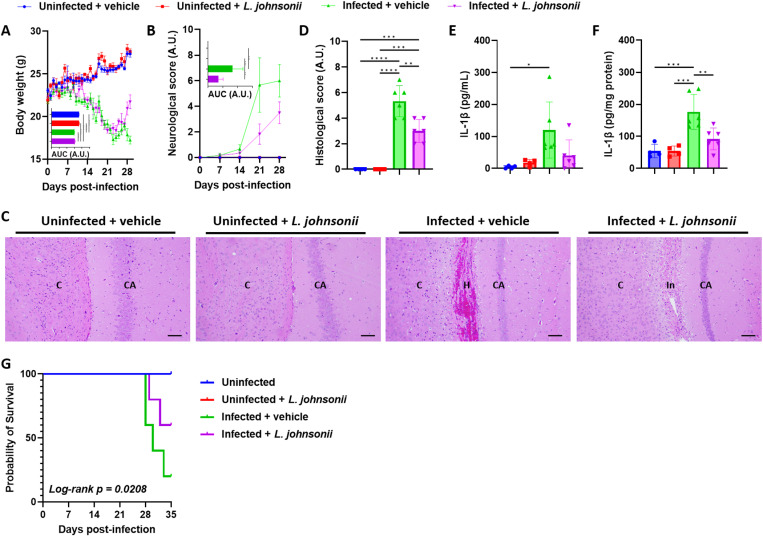
Inoculation of *Lactobacillus johnsonii* improves neuroangiostrongyliasis in mice. (A) The body weight of mice. (B) Neurological score of mice. (C) Representative H&E-stained section of the mouse brain. C, cerebral cortex; CA, cornu ammonis; H, hemorrhage; In, inflammatory infiltration. Scale bars correspond to 200 μm. (D) Histological score of the brain sections. (E-F) Serum (E) and brain (F) interleukin (IL)-1β levels. (G) Kaplan-Meier survival curves of *L. johnsonii*-inoculated mice, with or without *A. cantonensis* infection. For (A-F), ****n**** = 4 uninfected mice (with or without *L. johnsonii* inoculation) and ****n**** = 6 infected mice (with or without *L. johnsonii* inoculation); for (G), ****n**** = 5 mice in each group except ****n**** = 3 uninfected mice with *L. johnsonii* inoculation. Data in (A and B) are presented as mean ± S.E.M and (D-F) as mean ± S.D; area under curve (AUC) data are presented as mean ± S.D. A.U., arbitrary unit. * ***p*** < 0.05; ** ****p**** < 0.01; *** ****p**** < 0.001; and **** ****p**** < 0.0001. Significance determined by one-way ANOVA (A-F) or Log-rank test (G), with ****p**** < 0.05 considered significant.

To determine the possible role of *Lactobacillus* in angiostrongyliasis, *L. johnsonii* – which has been linked to an increase in *Lactobacillus* abundance (S3D Fig) and the disease (Fig 3E) and is known to have psychoactive effects [15] – was administered to *A. cantonensis*-infected mice. Compared with infected mice that were given water only, a repeated oral administration of *L. johnsonii* was associated with significantly improved changes in physiology and behavior. Body weight and neurological functions were also considerably improved in the infected mice with *L. johnsonii* inoculation (Fig 4A and 4B). While an extensive hemorrhage was accompanied by perivascular inflammatory infiltration in the brain of infected mice, mice fed with *L. johnsonii* revealed lesser inflammatory infiltration and meningoencephalitis (Fig 4C and 4D). As expected, *A. cantonensis* infection significantly induces serum and brain IL-1β (Fig 4E and 4F). Although serum IL-1β only non-significantly decreased in *L. johnsonii*-treated infected mice compared to vehicle-treated infected mice (Fig 4E), IL-1β levels in the brain of *L. johnsonii*-treated infected mice were significantly reduced (Fig 4F), suggesting a beneficial role of *L. johnsonii* in improving angiostrongyliasis. In accordance with these results, the survival rate of *L. johnsonii*-treated infected mice was improved compared to vehicle-treated infected mice (Fig 4G). Notably, we found that *L. johnsonii* treatment did not affect behavior, body weight, neurological functions, histopathology, or IL-1β levels in uninfected controls, compared to uninfected mice receiving vehicles.

### *Lactobacillus johnsonii* improves angiostrongyliasis by modulating the immune response

**Fig 5:**
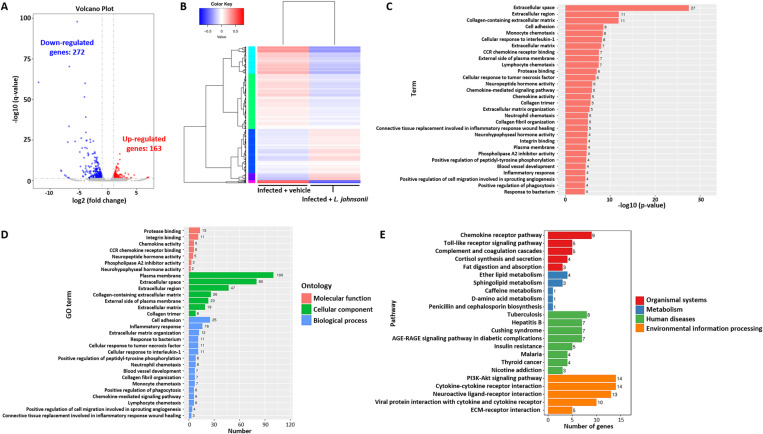
*Lactobacillus johnsonii* inoculation alters immune pathways in mice with neuroangiostrongyliasis. Transcriptomic analysis of the brain between *A. cantonensis-*infected mice with vehicle or *Lactobacillus johnsonii* inoculation. (A) Volcano plot of differentially expressed genes (DEGs) identified between vehicle-treated and *L. johnsonii*-treated, *A. cantonensis*-infected mice. Data are presented in log2 fold change of gene expression. Each dot represents a single gene. Grey dots represent non-significant genes, blue dots represent down-regulated DEGs, and red dots represent up-regulated DEGs. Significant DEGs are indicated as FC > 2 and FDR < 0.05. (B) Heatmap showing the 435 significant DEGs. Different color codes represent different clusters of genes with similar functions or participate in the same biological processes. Clustering was done with the Log_10_(FPKM + 1) values. (C) Gene ontology (GO) classifications based on significantly enriched GO terms. The x-axis shows the -log10(*p*-value) of each term. (D) GO classifications based on the number of DEGs in each GO category. The x-axis shows the number of DEGs. Only the top 30 most prominent GO categories are shown. Different color codes represent different GO categories. (E) KEGG enrichment histogram. The x-axis indicates the number of genes in each pathway term.

To determine the mechanism of how *L. johnsonii* improves the pathophysiology of angiostrongyliasis, a transcriptomic analysis of the brain tissue of vehicle-treated infected mice and *L. johnsonii*-treated infected mice was done. After filtering genes with low counts, 435 differentially expressed genes were identified, of which 272 were downregulated and 163 were upregulated in *L. johnsonii*-treated infected mice (Fig 5A and S6 Table). Clustering of these differential genes revealed that they may participate in similar biological processes (Fig 5B). Functional analysis of the gene ontology revealed enrichment in 38 terms (FDR-adjusted *p* value < 0.05), where 9 terms belong to the molecular function (MF) ontology, 8 terms belong to cellular component (CC) ontology, and 21 terms belong to biological process (BP) ontology (Fig 5C and S7 Table). When analyzed according to the number of enriched genes in each term, protease binding (GO:0002020), integrin binding (GO:0005178), and chemokine activity (GO:0008009) were most significantly enriched in the MF ontology. On the other hand, plasma membrane (GO:0005886), extracellular space (GO:0005615), and extracellular region (GO:0005576) were most enriched in the CC ontology, while cell adhesion (GO:0007155), inflammatory response (GO:0006954), and extracellular matrix organization (GO:0030198) were most enriched in the BP ontology (Fig 5D). Other immune-mediated processes such as chemokine activity and immune cell chemotaxis were also enriched in the BP ontology (Fig 5D).

To gain a deeper insight into the possible modulation by *L. johnsonii*, a KEGG analysis was performed. The analysis identified 23 enriched pathways (FDR-adjusted *p* value < 0.05), with 5 involved in the organismal systems level, 5 involved in the metabolism level, 8 involved in the human diseases level, and 5 involved in the environmental information processing level (Fig 5E and S8 Table). We next investigated pathways containing more than five differentially expressed genes, excluding the human disease level (as it is not relevant in this study). The identified pathways within the organismal systems level include chemokine signaling pathway (ko04062), toll-like receptor signaling pathway (ko04620), and complement and coagulation cascades (ko04610); and genes identified within these pathways are downregulated in response to *L. johnsonii* treatment in the context of *A. cantonensis* infection (S4A Fig). No pathways were identified within the metabolism level with more than five differentially expressed genes. Finally, all five pathways identified in the environmental information processing level have more than five differentially expressed genes and were related to immune modulation, which included PI3K-Akt signaling pathway (ko04151), cytokine-cytokine receptor interaction (ko04060), neuroactive ligand-receptor interaction (ko04080), viral protein interaction with cytokine and cytokine receptor (ko04061), and ECM-receptor interaction (ko04512). Similarly, all the genes annotated within these pathways were downregulated in the *L. johnsonii*-treated mice compared to vehicle-treated mice (S4B Fig). These results therefore suggested that *L. johnsonii* may improve angiostrongyliasis by modulating the immune response.

**Fig 6:**
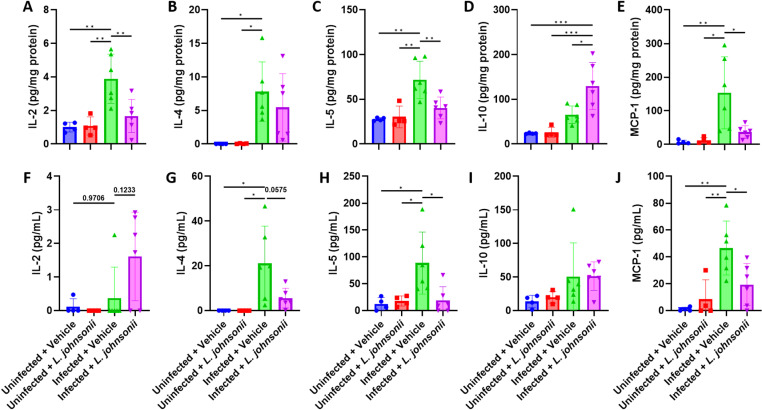
*Lactobacillus johnsonii* inoculation alters immune response in mice with neuroangiostrongyliasis. (A-E) Levels of IL-2 (A), IL-4 (B), IL-5 (C), IL-10 (D), and MCP-1 (E) in the mice brain. (F-J) Levels of IL-2 (E), IL-4 (F), IL-5 (G), IL-10 (H), and MCP-1 (I) in the mice serum. ****n**** = 4 uninfected mice (with or without *L. johnsonii* inoculation) and ****n**** = 6 infected mice (with or without *L. johnsonii* inoculation). Data are presented as mean ± S.D. * ****p**** < 0.05; ** ****p**** < 0.01; and *** ****p**** < 0.001. Significance as determined by one-way ANOVA, with ****p**** < 0.05 considered significant.

Therefore, we investigated the immune response that may play a role in *L. jonhsonii*-associated improvement. As expected, we identified higher IL-2, IL-4, IL-5, IL-10, and MCP-1 levels in the brains of infected mice compared to uninfected mice. When comparing between vehicle-treated infected mice and *L. johnsonii*-treated infected mice, we revealed that IL-2, IL-4, IL-5, and MCP-1 levels decreased in *L. johnsonii*-treated infected mice (Fig 6A-E), whereas IL-10 levels were further increased (Fig 6D). On the other hand, higher serum levels of IL-4, IL-5, and MCP-1 were identified in vehicle-treated infected mice compared to the uninfected mice, with levels decreased in *L. johnsonii*-treated infected mice compared to vehicle-treated infected mice (Fig 6F-J). Note that serum IL-10 was only non-significantly increased in infected mice, but it was not altered by *L. johnsonii* inoculation (Fig 6I). Serum IL-2, although not altered in vehicle-treated infected mice compared to uninfected mice, was non-significantly increased in *L. johnsonii*-treated infected mice compared to the other groups, suggesting a possible involvement of this cytokine (Fig 6F).

### The improvements of angiostrongyliasis by *Lactobacillus johnsonii* were not significantly related to microbial metabolites or gamma-aminobutyric acid

**Fig 7:**
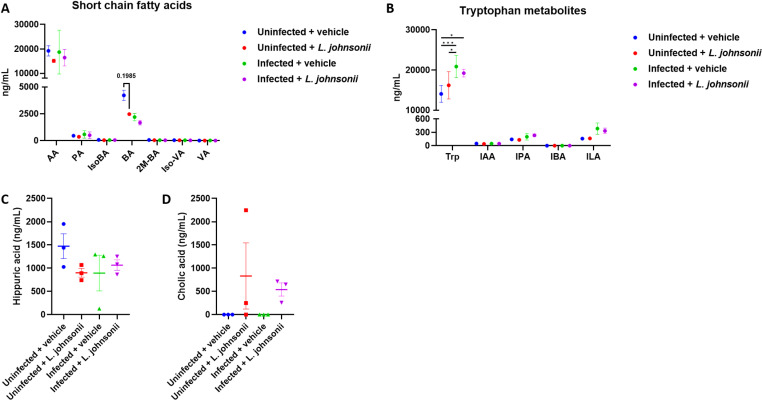
*Lactobacillus johnsonii* inoculation did not affect metabolites in mice with *Angiostrongylus cantonensis* infection. (A) Serum level of short-chain fatty acids. AA, acetic acid; PA, propionic acid; IsoBA, isobutyric acid; BA, butyric acid; 2M-BA, 2-methylbutyric acid; Iso-VA, isovaleric acid; VA, valeric acid. (B) Serum level of tryptophan metabolites. Trp, tryptophan; IAA, indole-3-acetic acid; IPA, indole-3-propionic acid; IBA, indole-3-butyric acid; ILA, indolelactic acid. (C) Serum level of hippuric acid. (D) Serum levels of cholic acid. Other bile acids, including ursodeoxycholic acid, chenodeoxycholic acid, deoxycholic acid, lithocholic acid, glycoursodeoxycholic acid, glycocholic acid, glycochenodeoxycholic acid, and glycodeoxycholic acid were not detected. ****n**** = 3 mice in each group. Values below the limit of detection are typed in as 0. Data are presented as mean ± S.E.M. * ****p**** < 0.05 and *** ****p**** < 0.001. Significance determined by one-way ANOVA, with ****p**** < 0.05 considered significant.

Currently, gut microbiome-related metabolites and their derivatives have been suggested as the major contributors to the gut-brain axis communication [16]; therefore, we intend to investigate whether improvements in angiostrongyliasis are also related to changes in metabolites. Important microbiome-related metabolites, including short-chain fatty acids, tryptophan metabolites, hippuric acids, and bile acids, were measured in the mice serum. However, of all the short-chain fatty acids investigated, only butyric acid levels were found to be altered between groups (though not statistically significant). Infected mice showed lower butyric acid levels compared to uninfected mice, and *L. johnsonii*-fed mice, with or without *A. cantonensis* infection, also showed suppressed butyric acid levels compared to vehicle-treated mice (Fig 7A). Regarding tryptophan metabolites, only tryptophan levels were significantly increased in infected mice compared to uninfected mice, but inoculation of *L. johnsonii* did not alter tryptophan levels. While indole-3-propionic acid (IPA) and indoleacetic acid (ILA) levels were also increased, although not statistically significant, in infected mice, we suggest a possible involvement of tryptophan metabolism in *A. cantonensis* infection; however, this metabolic pathway was not altered by *L. johnsonii* treatment (Fig 7B). Hippuric acid is a microbial metabolite found in the brain and has been shown to promote neurodevelopment including axonogenesis [16,17]. However, no changes were observed in hippuric acid levels between groups (Fig 7C). Lastly, different bile acids were measured in the serum, but only cholic acid were detected (Fig 7D). Only *L. johnsonii*-fed mice showed increased cholic acid levels, but they were not statistically significant (Fig 7D).

KEGG analysis, in addition to the immune-related response, also identified neuroactive ligand-receptor interaction (Fig 5D). By analyzing the differential gene set, we revealed a differential expression of genes related to gamma-aminobutyric acid (GABA) signaling, mostly downregulating in *Lactobacillus*-inoculated infected mice except for *Gabrb2* (S4A Fig). Protein-protein association network analysis also suggested a possible involvement of the GABAergic synapse (S4B Fig). However, although infected mice had slightly higher GABA levels than uninfected mice and *L. johnsonii*-fed infected mice had lower GABA levels than infected mice, they were not statistically significant (S4C Fig). These results suggested that although partially involved in the experiment conditions, microbial metabolites and GABA may not be the major reason for *L. johnsonii*-associated improvement.

### *Lactococcus lactis* lessens angiostrongyliasis symptoms

*Lactobacillus* is one of the main genera of lactic acid bacteria, which plays an important role in the nutritional value of foods and in maintaining a healthy gut microbiome [18]. As we observed the beneficial role of *Lactobacillus* in angiostrongyliasis, we wondered whether other lactic acid bacteria also have a similar effect. For this reason, we treated *A. cantonensis*-infected mice with *Lactococcus lactis*, another important lactic acid bacteria [18]. In agreement with our findings for *L. johnsonii*, treating infected mice with *L. lactis* revealed improved body weight (S5A Fig) and neurological functions (S5B Fig). Moreover, histopathological analysis revealed a lesser inflammatory response in *L. lactis*-treated infected mice than in vehicle-treated infected mice (S5C and S5D Fig). IL-1β levels in the brain (S5E Fig) and serum (S5F Fig) were also decreased in *L. lactis*-treated infected mice. Together, these results suggest that other lactic acid bacteria may also modulate the course of angiostrongyliasis and provide a survival benefit for *A. cantonensis*-infected mice.

## Discussion

The gut-brain axis has suggested a fundamental role of the gut microbiome in regulating neurological disorders, including the modulation of neuroinflammation and behavioral changes [16]. In neuroangiostrongyliasis, humans accidentally ingest L3-infected snails or L3-contaminated foods, which results in the migration of the larvae into the brain, causing inflammation. To investigate the role of the gut microbiome in neuroangiostrongyliasis, we used an oral infection model with BALB/c mice. Our study highlights the potential involvement of *L. johnsonii* in serving as a hub for modulating angiostrongyliasis. *Lactobacillus* was long considered a good bacterium and has therefore been commercialized in many probiotic products [19]. It shows good adherence to the intestinal epithelium and can positively or negatively influence innate and adaptive immunity [20,21].

In this study, we first identified an unchanged alpha diversity (Fig 2A-C) but differences in beta diversity (Fig 2D) between uninfected and infected mice. These findings suggest that the dominant species between the two groups are similar, while taxonomic changes occur within the groups. Currently, no study has investigated the involvement of gut microbiome in humans with *A. cantonensis* infection, but a study was done in the freshwater snail *Biomphalaria glabrata* and the terrestrial slug *Phillocaulis soleiformis*. It was shown that the alpha diversity in both *B. glabrata* and *P. soleiformis* was not significantly altered after *A. cantonensis* infection [22]. On the other hand, differences in beta diversity were observed between uninfected and infected *B. glabrata*. Family *Weeksellaceae* significantly increased in infected *B. glabrata,* while genus *Fluviicola* significantly decreased in infected *B. glabrata*. However, no *Lactobacillus* was identified in the microbiome of the snails [22]. Although snails differ from humans and mice in terms of microbiome composition and their role in the *A. cantonensis* life cycle, they exhibited outcomes similar to those in our study, including unchanged alpha diversity and altered beta diversity. This suggests that *A. cantonensis* may not impact the overall microbial diversity within the host but could alter the microbiome composition. On a phylum level, we identified a decrease in *Bacteroides* and an increase in *Firmicutes* in infected mice (Fig 2E and S3 Table). *Bacteroides* are considered beneficial for regulating inflammation [23–25], while *Firmicutes* have been linked to inflammation [26,27]. This corroborates our findings in *Angiostrongylus*-induced neuroinflammation.

An increase in the abundance of *Lactobacillus* in *A. cantonensis*-infected mice was found, which was correlated with the severity of the disease (Fig 3). This leads us to hypothesize that *Lactobacillus* may have an unknown negative effect. However, attempts to inoculate *L. johnsonii* into *A. cantonensis*-infected mice surprisingly revealed an improvement in the disease and better survival (Fig 4). An increase in IL-2, IL-4, and IL-5 was expected during *A. cantonensis* infection (Fig 6A-C). While IL-2 is involved in T-cell activation, IL-4 and IL-5 play roles in eosinophil activation [28–30]. These cytokines are crucial for protection against the parasite or, in the case of an excessive response, the pathogenesis of angiostrongyliasis. The increase of MCP-1 (Fig 6E) was also anticipated during infection, as it promotes the influx of inflammatory cells into the CNS [31]. Further analysis revealed a regulated immune response, possibly associated with *L. johnsonii*, leading to better disease outcomes (Figs 5 and 6). Previously, several literatures have reported an immune regulatory effect of *Lactobacilli*, mainly on upregulating regulatory T (Treg) cells [32–35]. As most effector T cells, including T helper 1 (Th1) and Th2 cells, are regulated by Treg cells [36], the suppressed Th1 and Th2 response, as indicated by decreased IL-2, IL-4, and IL-5 levels, by *L. johnsonii* inoculation (Fig 6A-C) may suggest a role of Treg cells in improving angiostrongyliasis. As expected, we found an increased IL-10 level in the brain of *L. johnsonii*-treated, *A. cantonensis*-infected mice (Fig 6D). IL-10, as classified as a Treg cytokine, exerts a suppressive function on Th1 and Th2 cells [37]. IL-10 has also been shown to be secreted by almost all of the innate and adaptive immune cells including macrophages, B cells, cytotoxic T cells, Th1 cells, Th2 cells, and Th17 cells, suggesting diverse targets and functions of IL-10 [38–41]. Previously, *Lactobacilli* have been reported to induce IL-10-mediated Treg cell functions [42–45], including species identified in this study (Fig 3D-F). However, several other species such as *Lactobacillus rhamnosus* LA68 [46], *Lactobacillus brevis* HY7401 [47], *Lactobacillus rhamnosus* GG [48], and *Lactobacillus fermentum* CJL-112 [49] have been shown to suppress IL-10 to achieve their beneficial effects. Therefore, the mechanism of how the multifaceted IL-10 plays a role in the beneficial effect of *Lactobacillus* against angiostrongyliasis may require further analysis. We also analyzed the level of the inflammatory chemokine MCP-1. MCP-1 leads to the migration and infiltration of monocytes and macrophages [50], inculpating different neuroinflammatory disorders [51]. Therefore, the inhibition of MCP-1 by *L. johnsonii* is reasoned for the improved CNS inflammation in infected mice. Similar findings were observed in the serum levels of IL-4, IL-5, and MCP-1 (Fig 6G, 6H and 6J), further confirming the involvement of these cytokines and chemokines. To noted, serum IL-2 levels differed from those observed in the brain and were further elevated in *L. johnsonii*-treated infected mice (Fig 6F). This difference could be due to different regulatory mechanisms between the brain (local) and systemic immune activation. While both blood-derived IL-2 and brain-derived IL-2 can enhance immune activation and modulate neuroinflammation [29,52,53], not all of the blood-derived IL-2 can cross the blood-brain barrier (BBB) [54]. The inability of IL-2, induced by *L. johnsonii* in the periphery, to cross the BBB may resulted from a regulatory mechanism to prevent excessive neuroinflammation. In conclusion, these results support the idea that although intestinal *L. johnsonii* correlated with the disease progression of neuroangiostrongyliasis, these bacteria are more likely to try to modulate the dysregulated immunity to fight against the disease.

We further investigate the possible microbial metabolites that may play a role in the gut-brain axis; however, most of the investigated metabolites were not altered by either *A. cantonensis* or *L. johnsonii* (Fig 7). We identified a decreased butyric acid among all the tested short-chain fatty acids across all experimental groups. Butyric acid has been shown to maintain blood-brain barrier integrity [55,56]; therefore, the decrease in butyric acid levels in *A. cantonensis*-infected mice may explain the possible pathogenesis of the disease and the mechanism of immune cell trafficking into the brain (Figs 1C and 7A). However, inoculation of *L. johnsonii* did not restore butyric acid levels. On the other hand, tryptophan, IPA, and ILA levels were increased, although not always showing statistical significance, in the infected mice (Fig 7B). It was shown that these tryptophan metabolites in the indole pathway can positively or negatively regulate immunity by modulating the aryl hydrocarbon receptors (AHR) [57,58]. AHR activation is thought to contribute to anti-inflammation [58]; therefore, the presence of tryptophan metabolites in the infected mice can be considered compensation to reduce neuroinflammation caused by the parasite. Similarly, *L. johnsonii* inoculation did not affect these metabolites. Finally, we found that *L. johnsonii* increased serum cholic acid levels regardless of *A. cantonensis* infection (Fig 7D). Cholic acid is one of the major bile acids produced by the liver. Previously, *Lactobacillus* has been shown to induce spontaneous accumulation of cholic acids, driven by their transmembrane proton gradient [59] and their bile salt deconjugation ability [60,61]. This action serves as a way to alter bile acid signaling [62], thereby leading to the host’s metabolic control and immune homeostasis [63,64]. We therefore hypothesize that the induction of cholic acid by *L. johnsonii* may be one of the reasons for the immune restoration observed in *A. cantonensis*-infected mice, which, at that time, is characterized by an immune imbalance against the parasite. However, this was not observed in the uninfected mice, as they exhibited normal immunity. On the other hand, RNA-seq suggested a potential involvement of GABA signaling in *L. johnsonii* treatment (Fig 5). GABA is the primary inhibitory neurotransmitter in the CNS, and disturbances in GABA metabolism or GABAergic inhibition can lead to neurological disorders [65]. We observed a slight increase in GABA levels in *A. cantonensis*-infected mice (S5 Fig), suggesting a disturbance in GABAergic signaling. Following *L. johnsonii* treatment, GABA levels reverted to levels comparable to those of uninfected mice, indicating an improvement in the disease. However, the changes observed did not reach statistical significance. Given the limited sample size in this study, we cannot exclude the possibility that microbial metabolites and GABAergic signaling may also be modulated by *L. johnsonii* and contribute to improving angiostrongyliasis. Therefore, future experiments with a larger sample size would be beneficial in elucidating the underlying mechanism.

We also inoculated *A. cantonensis*-infected mice with another lactic acid bacteria, *L. lactis*, which revealed similar beneficial effects (S5 Fig). *L. lactis* has been shown to process anti-inflammatory and immunomodulatory effects [66]; for example, oral gavage of *L. lactis* HFY14 has ameliorated antibiotic-induced brain inflammation [67]. Similar to *Lactobacillus*, *Lactococcus* can induce IL-10 to reduce inflammatory immune response [10,68]. Metabolites from both bacteria have improved innate and acquired immunity, preventing allergies, inflammation, and even pathogen invasion [69]. These findings further highlight the potential of using *Lactobacillus* or a complex of lactic acid bacteria as a potential treatment against neuroangiostrongyliasis. Moreover, *L. lactis* used in this study was isolated from a commercial probiotic product; our results presented here, therefore, provide insights into the potential application of commercial products in treating neuroangiostrongyliasis.

We note that our study mainly focuses on *L. johnsonii*, while other *Lactobacillus* species were also found to be differentially altered in *A. cantonensis*-infected mice (Fig 3). Future studies may investigate the role of different *Lactobacillus* species and other possible bacteria, such as *Muribaculaceae*, in neuroangiostrongyliasis. Additionally, the animal used in this study had a normal microbiome, which may have influenced the results of bacterial inoculation. Future studies using antibiotic pre-treatment to clear the gut microbiome or employing germ-free mice could offer a more comprehensive understanding of the role of *Lactobacillus* in neuroangiostrongyliasis. In addition, while RNA-seq data suggested that *L. johnsonii* may inhibit upstream TLR signaling in angiostrongyliasis (Fig 5E), with a downregulation of the TLR-7 gene observed (S4A Fig), qPCR validation did not confirm a decrease in gene expression (S4C Fig). Although TLR-7 has been linked with neuroinflammation [70], other TLRs may also contribute to the beneficial effects of *L. johnsonii* in angiostrongyliasis, as similar effects have been observed in other diseases [71,72]. Therefore, future studies focusing on the host response may be helpful in clarifying this.

This is one of the first studies investigating the gut microbiome in mice with *A. cantonensis* infection. This study provided a preliminary explanation from the microbiome point-of-view of the pathogenesis of angiostrongyliasis and how the microbiome defends against *A. cantonensis*. Nevertheless, using different *Lactobacillus* species or bacterial strains may serve as a continuous incentive for future studies.

## Supporting information

S1 Table Neurological scoring system. (XLSX)

S2 Table Primer pairs used in this study. (XLSX)

S3 Table Relative abundance of the microbiome. (XLSX)

S4 Table Unpaired T-test analysis of the microbiome abundance. (XLSX)

S5 Table LEfSe and STAMP analysis of enriched microbiome. (XLSX)

S6 Table Significant annotation of the transcriptome. (XLS)

S7 Table GO analysis of the significant transcriptome. (XLSX)

S8 Table KEGG analysis of the significant transcriptome. (XLSX)

S1 Data 16S sequencing information and alignment data of *Lactococcus lactis* used in this study. (DOCX)

S1 Fig Representative image of bacterial culture of *Lactobacillus johnsonii* and *Lactococcus lactis* on blood agar plate. (TIF)

S2 Fig (A) LEfSe (linear discriminant analysis effect size) analysis showing the classification of species with significant effects in uninfected and *A. cantonensis*-infected mice. The horizontal coordinate shows the logarithmic LDA scores for each taxonomic unit. The longer lengths indicate the more significant differences in the classification. The default setting was LDA effect size > 3 and *p*-value < 0.05. (B) STAMP analysis at the genus levels showing significant differences in the microbiome abundance between uninfected and *A. cantonensis*-infected mice. The left figure shows the percentage of mean proportion of bacterial strains in the two groups; the right figure shows the percentage of difference of mean proportion of bacterial genera within the 95% confidence interval; *p*-values are shown on the right. *n* = 6 mice in both groups. Significance determined by Welch’s t-test, with *p* < 0.05 considered significant.
(TIF)

S3 Fig Scatter plot of Pearson’s correlation coefficienct (*r*) between *Lactobacillus murinus* (A), *Lactobacillus johnsonii* (B), and *Lactobacillus rueteri* (C) abundance and neurological score or histological score. (D) Pearson’s correlation matrix between different variables. Pearson correlation coefficient values (*r*) are shaded with different color codes: positive correlations are from white to blue on the color scale, and negative correlations are from white to red. No negative correlation was seen between these variables. Bacterial abundance was qPCR data. A.U., arbitrary unit. *n* = 6 mice in the uninfected and infected group.
(TIF)

S4 Fig KEGG analysis of pathways containing five or more differentially expressed genes. (A) Identified pathways within the organismal systems level including chemokine signaling pathway (ko04062), toll-like receptor signaling pathway (ko04620), and complement and coagulation cascades (ko04610). (B) Identified pathways within the environmental information processing level including PI3K-Akt signaling pathway (ko04151), cytokine-cytokine receptor interaction (ko04060), neuroactive ligand-receptor interaction (ko04080), viral protein interaction with cytokine and cytokine receptor (ko04061), and ECM-receptor interaction (ko04512). AC, brain from *Angiostrongylus cantonensis*-infected mice; ACLJ, brain from *Lactobacillus johnsonii*-treated, *A. cantonensis*-infected mice. Gene expression levels are shaded with different colors on a color scale from green (lower expression) to red (higher expression). (C) qPCR validation of selected genes. Data are presented as mean ± S.D. *n* = 6 mice in both groups. Significance determined by unpaired T-test, with *p* < 0.05 considered significant.
(TIF)

S5 Fig (A) Differences of gamma-aminobutyric acid (GABA)-related transcriptome between vehicle-treated *Angiostrongylus cantonensis*-infected mice and *Lactobacillus johnsonii*-treated, *A.
* ***cantonensis*-infected mice.** (B) Network interaction map showing potential protein-protein interaction of the identified transcriptome in (A). The analysis was carried out with the publically available STRING database (https://string-db.org/; version 12.0). (C) Serum levels of GABA. *n* = 4 uninfected mice (with or without *L. johnsonii* inoculation) and *n* = 6 infected mice (with or without *L. johnsonii* inoculation). Data are presented as mean ± S.E.M. Significance determined by (A) false discovery rate (FDR) or (C) one-way ANOVA with Tukey’s honest significant difference test. FDR < 0.05 is considered significant.
(TIF)

S6 Fig Inoculation of *Lactobacillus lactis* improves neuroangiostrongyliasis in mice. (A) The body weight of mice. (B) Neurological score of mice. (C) Representative H&E-stained section of the mouse brain. Scale bars correspond to 200 μm. (D) Histological score of the brain sections. (E-F) Serum (E) and brain (F) interleukin (IL)-1β levels. *n* = 3 mice in each group. Data in (A and B) are presented as mean ± S.E.M and (D-F) as mean ± S.D; area under curve (AUC) data are presented as mean ± S.D. A.U., arbitrary unit. Significance determined by unpaired T-test, with *p* < 0.05 considered significant.
(TIF)
