# Supplementary material for: Intestinal Lactobacillus johnsonii protects against neuroangiostrongyliasis in BALB/c mice through modulation of immune response
Source: PLoS Negl Trop Dis. 2025 Apr 8;19(4):e0012977. doi: 10.1371/journal.pntd.0012977 (PMC11978024; doi:10.1371/journal.pntd.0012977)
Supplement: S6 Table — (XLS) [file pntd.0012977.s006.docx]

**S1 Data. Lactococcus 16S sequence and alignment**

TGCAAGTTGAGCGCTGAAGGTTGGTACTTGTACCAACTGGATGAGCAGCGAACGGGTGAGTAACGCGTGGGGAATCTGCCTTTGAGCGGGGGACAACATTTGGAAACGAATGCTAATACCGCATAAAAACTTTAAACACAAGTTTTAAGTTTGAAAGATGCAATTGCATCACTCAAAGATGATCCCGCGTTGTATTAGCTAGTTGGTGAGGTAAAGGCTCACCAAGGCGATGATACATAGCCGACCTGAGAGGGTGATCGGCCACATTGGGACTGAGACACGGCCCAAACTCCTACGGGAGGCAGCAGTAGGGAATCTTCGGCAATGGACGAAAGTCTGACCGAGCAACGCCGCGTGAGTGAAGAAGGTTTTCGGATCGTAAAACTCTGTTGGTAGAGAAGAACGTTGGTGAGAGTGGAAAGCTCATCAAGTGACGGTAACTACCCAGAAAGGGACGGCTAACTACGTGCCAGCAGCCGCGGTAATACGTAGGTCCCGAGCGTTGTCCGGATTTATTGGGCGTAAAGCGAGCGCAGGTGGTTTATTAAGTCTGGTGTAAAAGGCAGTGGCTCAACCATTGTATGCATTGGAAACTGGTAGACTTGAGTGCAGGAGAGGAGAGTGGAATTCCATGTGTAGCGGTGAAATGCGTAGATATATGGAGGAACACCGGTGGCGAAAGCGGCTCTCTGGCCTGTAACTGACACTGAGGCTCGAAAGCGTGGGGAGCAAACAGGATTAGATACCCTGGTAGTCCACGCCGTAAACGATGAGTGCTAGATGTAGGGAGCTATAAGTTCTCTGTATCGCAGCTAACGCAATAAGCACTCCGCCTGGGGAGTACGACCGCAAGGTTGAAACTCAAAGGAATTGACGGGGGCCCGCACAAGCGGTGGAGCATGTGGTTTAATTCGAAGCAACGCGAAGAACCTTACCAGGTCTTGACATACTCGTGCTATTCCTAGAGATAGGAAGTTCCTTCGGGACACGGGATACAGGTGGTGCATGGTTGTCGTCAGCTCGTGTCGTGAGATGTTGGGTTAAGTCCCGCAACGAGCGCAACCCCTATTGTTAGTTGCCATCATTAAGTTGGGCACTCTAACGAGACTGCCGGTGATAAACCGGAGGAAGGTGGGGATGACGTCAAATCATCATGCCCCTTATGACCTGGGCTACACACGTGCTACAATGGATGGTACAACGAGTCGCGAGACAGTGATGTTTAGCTAATCTCTTAAAACCATTCTCAGTTCGGATTGTAGGCTGCAACTCGCCTACATGAAGTCGGAATCGCTAGTAATCGCGGATCAGCACGCCGCGGTGAATACGTTCCCGGGCCTTGTACACACCGCCCGTCACACCACGGGAGTTGGGAGTACCCGAAGTAGGTTGCCTAACCGCAAGGAGGGCGCTTCCTAAGGTAAGACCGATGACTGGGGTGAAGTCGTAACAG

| **Description** | **Scientific Name** | **Max Score** | **Total Score** | **Query Cover** | **E value** | **Per. ident** | **Acc. Len** | **Accession** |
| --- | --- | --- | --- | --- | --- | --- | --- | --- |
| Lactococcus sp. 003.41 16S ribosomal RNA gene, partial sequence | Lactococcus sp. 003.41 | 2684 | 2684 | 100% | 0 | 100 | 1487 | [KU639597.1](https://www.ncbi.nlm.nih.gov/nucleotide/KU639597.1?report=genbank&log$=nucltop&blast_rank=1&RID=FYETSPVH016) |
| Lactococcus lactis strain FAM 17919 chromosome, complete genome | Lactococcus lactis | 2682 | 16089 | 99% | 0 | 100 | 2455104 | [CP093959.1](https://www.ncbi.nlm.nih.gov/nucleotide/CP093959.1?report=genbank&log$=nucltop&blast_rank=2&RID=FYETSPVH016) |
| Lactococcus lactis subsp. lactis strain C10, complete genome | Lactococcus lactis subsp. lactis | 2682 | 13399 | 99% | 0 | 100 | 2335639 | [CP015898.1](https://www.ncbi.nlm.nih.gov/nucleotide/CP015898.1?report=genbank&log$=nucltop&blast_rank=3&RID=FYETSPVH016) |
| Lactococcus lactis subsp. lactis strain UC063 chromosome, complete genome | Lactococcus lactis subsp. lactis | 2682 | 16094 | 99% | 0 | 100 | 2392933 | [CP015905.2](https://www.ncbi.nlm.nih.gov/nucleotide/CP015905.2?report=genbank&log$=nucltop&blast_rank=4&RID=FYETSPVH016) |
| Lactococcus lactis strain P-1 chromosome, complete genome | Lactococcus lactis | 2682 | 16089 | 99% | 0 | 100 | 2568009 | [CP118738.1](https://www.ncbi.nlm.nih.gov/nucleotide/CP118738.1?report=genbank&log$=nucltop&blast_rank=5&RID=FYETSPVH016) |
| Lactococcus lactis strain 2B-9 chromosome, complete genome | Lactococcus lactis | 2682 | 16094 | 99% | 0 | 100 | 2623332 | [CP157500.1](https://www.ncbi.nlm.nih.gov/nucleotide/CP157500.1?report=genbank&log$=nucltop&blast_rank=6&RID=FYETSPVH016) |
| Lactococcus lactis strain LB7 chromosome, complete genome | Lactococcus lactis | 2682 | 16089 | 99% | 0 | 100 | 2603291 | [CP117409.1](https://www.ncbi.nlm.nih.gov/nucleotide/CP117409.1?report=genbank&log$=nucltop&blast_rank=7&RID=FYETSPVH016) |
| Lactococcus lactis strain LLKS5 16S ribosomal RNA gene, partial sequence | Lactococcus lactis | 2682 | 2682 | 99% | 0 | 100 | 1508 | [MK977611.1](https://www.ncbi.nlm.nih.gov/nucleotide/MK977611.1?report=genbank&log$=nucltop&blast_rank=8&RID=FYETSPVH016) |
| Lactococcus lactis strain 2B-1 chromosome, complete genome | Lactococcus lactis | 2682 | 16094 | 99% | 0 | 100 | 2623330 | [CP157495.1](https://www.ncbi.nlm.nih.gov/nucleotide/CP157495.1?report=genbank&log$=nucltop&blast_rank=9&RID=FYETSPVH016) |
| Lactococcus lactis strain SampleD1_2 chromosome, complete genome | Lactococcus lactis | 2682 | 16048 | 99% | 0 | 100 | 2459127 | [CP121454.2](https://www.ncbi.nlm.nih.gov/nucleotide/CP121454.2?report=genbank&log$=nucltop&blast_rank=10&RID=FYETSPVH016) |
| Lactococcus lactis ID-5 DNA, complete genome | Lactococcus lactis | 2682 | 16094 | 99% | 0 | 100 | 2489582 | [AP025701.1](https://www.ncbi.nlm.nih.gov/nucleotide/AP025701.1?report=genbank&log$=nucltop&blast_rank=11&RID=FYETSPVH016) |
| Lactococcus sp. strain Y1-B-3 16S ribosomal RNA gene, partial sequence | Lactococcus sp. | 2682 | 2682 | 99% | 0 | 100 | 1497 | [OK272203.1](https://www.ncbi.nlm.nih.gov/nucleotide/OK272203.1?report=genbank&log$=nucltop&blast_rank=12&RID=FYETSPVH016) |
| Lactococcus lactis isolate Lactococcus lactis CIRM-BIA2553 genome assembly, chromosome: 1 | Lactococcus lactis | 2682 | 16094 | 99% | 0 | 100 | 2400577 | [OZ061291.1](https://www.ncbi.nlm.nih.gov/nucleotide/OZ061291.1?report=genbank&log$=nucltop&blast_rank=13&RID=FYETSPVH016) |
| Lactococcus lactis strain SL(6) 16S ribosomal RNA gene, partial sequence | Lactococcus lactis | 2682 | 2682 | 99% | 0 | 100 | 1539 | [HQ591349.1](https://www.ncbi.nlm.nih.gov/nucleotide/HQ591349.1?report=genbank&log$=nucltop&blast_rank=14&RID=FYETSPVH016) |
| Lactococcus lactis subsp. lactis strain 275, complete genome | Lactococcus lactis subsp. lactis | 2682 | 16083 | 99% | 0 | 100 | 2495882 | [CP015897.1](https://www.ncbi.nlm.nih.gov/nucleotide/CP015897.1?report=genbank&log$=nucltop&blast_rank=15&RID=FYETSPVH016) |
| Lactococcus lactis subsp. lactis strain I.1.1 chromosome, complete genome | Lactococcus lactis subsp. lactis | 2682 | 16094 | 99% | 0 | 100 | 2459089 | [CP069223.2](https://www.ncbi.nlm.nih.gov/nucleotide/CP069223.2?report=genbank&log$=nucltop&blast_rank=16&RID=FYETSPVH016) |
| Lactococcus lactis subsp. lactis strain IBB109 chromosome, complete genome | Lactococcus lactis subsp. lactis | 2682 | 16094 | 99% | 0 | 100 | 2344660 | [CP087600.1](https://www.ncbi.nlm.nih.gov/nucleotide/CP087600.1?report=genbank&log$=nucltop&blast_rank=17&RID=FYETSPVH016) |
| Lactococcus lactis subsp. lactis strain UL021 chromosome, complete genome | Lactococcus lactis subsp. lactis | 2682 | 16094 | 99% | 0 | 100 | 2496922 | [CP070263.2](https://www.ncbi.nlm.nih.gov/nucleotide/CP070263.2?report=genbank&log$=nucltop&blast_rank=18&RID=FYETSPVH016) |
| Lactococcus lactis strain EP2 chromosome, complete genome | Lactococcus lactis | 2682 | 16094 | 99% | 0 | 100 | 2348459 | [CP115479.1](https://www.ncbi.nlm.nih.gov/nucleotide/CP115479.1?report=genbank&log$=nucltop&blast_rank=19&RID=FYETSPVH016) |
| Lactococcus lactis strain JNU 534 chromosome, complete genome | Lactococcus lactis | 2682 | 16094 | 99% | 0 | 100 | 2443687 | [CP095737.1](https://www.ncbi.nlm.nih.gov/nucleotide/CP095737.1?report=genbank&log$=nucltop&blast_rank=20&RID=FYETSPVH016) |
| Lactococcus lactis DCR3-2 gene for 16S rRNA, partial sequence | Lactococcus lactis | 2682 | 2682 | 99% | 0 | 100 | 1547 | [LC819332.1](https://www.ncbi.nlm.nih.gov/nucleotide/LC819332.1?report=genbank&log$=nucltop&blast_rank=21&RID=FYETSPVH016) |
| Lactococcus lactis subsp. lactis CV56, complete genome | Lactococcus lactis subsp. lactis CV56 | 2682 | 16089 | 99% | 0 | 100 | 2399458 | [CP002365.1](https://www.ncbi.nlm.nih.gov/nucleotide/CP002365.1?report=genbank&log$=nucltop&blast_rank=22&RID=FYETSPVH016) |
| Lactococcus lactis subsp. lactis strain IBB417 chromosome, complete genome | Lactococcus lactis subsp. lactis | 2682 | 16094 | 99% | 0 | 100 | 2380344 | [CP087699.1](https://www.ncbi.nlm.nih.gov/nucleotide/CP087699.1?report=genbank&log$=nucltop&blast_rank=23&RID=FYETSPVH016) |
| 3_Tms_b3v08 | Timema monikensis | 2682 | 2682 | 99% | 0 | 100 | 180949 | [OB794246.1](https://www.ncbi.nlm.nih.gov/nucleotide/OB794246.1?report=genbank&log$=nucltop&blast_rank=24&RID=FYETSPVH016) |
| Lactococcus lactis subsp. lactis strain 267 chromosome, complete genome | Lactococcus lactis subsp. lactis | 2682 | 16094 | 99% | 0 | 100 | 2447408 | [CP032058.2](https://www.ncbi.nlm.nih.gov/nucleotide/CP032058.2?report=genbank&log$=nucltop&blast_rank=25&RID=FYETSPVH016) |
| Lactococcus lactis subsp. lactis strain SCC43K 16S ribosomal RNA gene, partial sequence | Lactococcus lactis subsp. lactis | 2682 | 2682 | 99% | 0 | 100 | 1510 | [AY626141.1](https://www.ncbi.nlm.nih.gov/nucleotide/AY626141.1?report=genbank&log$=nucltop&blast_rank=26&RID=FYETSPVH016) |
| Lactococcus lactis strain FDAARGOS_1064 chromosome, complete genome | Lactococcus lactis | 2682 | 16065 | 99% | 0 | 100 | 2399760 | [CP065984.1](https://www.ncbi.nlm.nih.gov/nucleotide/CP065984.1?report=genbank&log$=nucltop&blast_rank=27&RID=FYETSPVH016) |
| Lactococcus lactis subsp. lactis strain Lor-MGB-YQ-2(1) 16S ribosomal RNA gene, partial sequence | Lactococcus lactis subsp. lactis | 2682 | 2682 | 99% | 0 | 100 | 1510 | [KC754747.1](https://www.ncbi.nlm.nih.gov/nucleotide/KC754747.1?report=genbank&log$=nucltop&blast_rank=28&RID=FYETSPVH016) |
| Uncultured bacterium clone 8C2-113 16S ribosomal RNA gene, partial sequence | uncultured bacterium | 2682 | 2682 | 99% | 0 | 100 | 1501 | [KJ424426.1](https://www.ncbi.nlm.nih.gov/nucleotide/KJ424426.1?report=genbank&log$=nucltop&blast_rank=29&RID=FYETSPVH016) |
| Lactococcus lactis strain DRD-8 chromosome | Lactococcus lactis | 2682 | 2682 | 99% | 0 | 100 | 2251213 | [CP133258.1](https://www.ncbi.nlm.nih.gov/nucleotide/CP133258.1?report=genbank&log$=nucltop&blast_rank=30&RID=FYETSPVH016) |
| Lactococcus lactis strain BIM B-1834 chromosome, complete genome | Lactococcus lactis | 2682 | 16089 | 99% | 0 | 100 | 2432875 | [CP157287.1](https://www.ncbi.nlm.nih.gov/nucleotide/CP157287.1?report=genbank&log$=nucltop&blast_rank=31&RID=FYETSPVH016) |
| Lactococcus lactis subsp. lactis gene for 16S rRNA, partial sequence, strain: NIAI 527 | Lactococcus lactis subsp. lactis | 2682 | 2682 | 99% | 0 | 100 | 1499 | [AB100795.1](https://www.ncbi.nlm.nih.gov/nucleotide/AB100795.1?report=genbank&log$=nucltop&blast_rank=32&RID=FYETSPVH016) |
| Lactococcus lactis subsp. lactis strain A12 genome assembly, chromosome: 1 | Lactococcus lactis subsp. lactis | 2682 | 16033 | 99% | 0 | 100 | 2603898 | [LT599049.1](https://www.ncbi.nlm.nih.gov/nucleotide/LT599049.1?report=genbank&log$=nucltop&blast_rank=33&RID=FYETSPVH016) |
| Lactococcus lactis strain LLKS7 16S ribosomal RNA gene, partial sequence | Lactococcus lactis | 2682 | 2682 | 99% | 0 | 100 | 1508 | [MK977613.1](https://www.ncbi.nlm.nih.gov/nucleotide/MK977613.1?report=genbank&log$=nucltop&blast_rank=34&RID=FYETSPVH016) |
| Lactococcus lactis strain DRD-23 chromosome | Lactococcus lactis | 2682 | 2682 | 99% | 0 | 100 | 2251214 | [CP133257.1](https://www.ncbi.nlm.nih.gov/nucleotide/CP133257.1?report=genbank&log$=nucltop&blast_rank=35&RID=FYETSPVH016) |
| Lactococcus lactis strain AFS020609 16S ribosomal RNA gene, partial sequence | Lactococcus lactis | 2682 | 2682 | 99% | 0 | 100 | 1539 | [OP986117.1](https://www.ncbi.nlm.nih.gov/nucleotide/OP986117.1?report=genbank&log$=nucltop&blast_rank=36&RID=FYETSPVH016) |
| Lactococcus lactis subsp. lactis strain CICC6016 16S ribosomal RNA gene, complete sequence | Lactococcus lactis subsp. lactis | 2682 | 2682 | 99% | 0 | 100 | 1480 | [DQ171717.1](https://www.ncbi.nlm.nih.gov/nucleotide/DQ171717.1?report=genbank&log$=nucltop&blast_rank=37&RID=FYETSPVH016) |
| Lactococcus lactis subsp. lactis strain UC06 chromosome, complete genome | Lactococcus lactis subsp. lactis | 2682 | 16094 | 99% | 0 | 100 | 2571403 | [CP015902.2](https://www.ncbi.nlm.nih.gov/nucleotide/CP015902.2?report=genbank&log$=nucltop&blast_rank=38&RID=FYETSPVH016) |
| Lactococcus lactis subsp. lactis gene for 16S rRNA, partial sequence, strain: Ni185 | Lactococcus lactis subsp. lactis | 2682 | 2682 | 99% | 0 | 100 | 1508 | [AB601163.1](https://www.ncbi.nlm.nih.gov/nucleotide/AB601163.1?report=genbank&log$=nucltop&blast_rank=39&RID=FYETSPVH016) |
| Lactococcus lactis MB52 gene for 16S ribosomal RNA, partial sequence | Lactococcus lactis | 2682 | 2682 | 99% | 0 | 100 | 1508 | [LC434018.1](https://www.ncbi.nlm.nih.gov/nucleotide/LC434018.1?report=genbank&log$=nucltop&blast_rank=40&RID=FYETSPVH016) |
| Uncultured Lactococcus sp. clone AV_5N-C11 16S ribosomal RNA gene, partial sequence | uncultured Lactococcus sp. | 2682 | 2682 | 99% | 0 | 100 | 1513 | [EU341204.1](https://www.ncbi.nlm.nih.gov/nucleotide/EU341204.1?report=genbank&log$=nucltop&blast_rank=41&RID=FYETSPVH016) |
| Lactococcus lactis subsp. lactis strain WM1 chromosome, complete genome | Lactococcus lactis subsp. lactis | 2682 | 16094 | 99% | 0 | 100 | 2446589 | [CP032500.2](https://www.ncbi.nlm.nih.gov/nucleotide/CP032500.2?report=genbank&log$=nucltop&blast_rank=42&RID=FYETSPVH016) |
| Lactococcus lactis strain IFLLLBEK1 chromosome, complete genome | Lactococcus lactis | 2682 | 16094 | 99% | 0 | 100 | 2498656 | [CP169315.1](https://www.ncbi.nlm.nih.gov/nucleotide/CP169315.1?report=genbank&log$=nucltop&blast_rank=43&RID=FYETSPVH016) |
| Lactococcus lactis strain SRCM103457 chromosome | Lactococcus lactis | 2682 | 16094 | 99% | 0 | 100 | 2458204 | [CP035757.1](https://www.ncbi.nlm.nih.gov/nucleotide/CP035757.1?report=genbank&log$=nucltop&blast_rank=44&RID=FYETSPVH016) |
| Lactococcus lactis strain GZC156 16S ribosomal RNA gene, partial sequence | Lactococcus lactis | 2682 | 2682 | 99% | 0 | 100 | 1511 | [MW898657.1](https://www.ncbi.nlm.nih.gov/nucleotide/MW898657.1?report=genbank&log$=nucltop&blast_rank=45&RID=FYETSPVH016) |
| Lactococcus lactis strain LAC460 chromosome, complete genome | Lactococcus lactis | 2682 | 16094 | 99% | 0 | 100 | 2426597 | [CP059048.1](https://www.ncbi.nlm.nih.gov/nucleotide/CP059048.1?report=genbank&log$=nucltop&blast_rank=46&RID=FYETSPVH016) |
| Lactococcus lactis strain E114 16S ribosomal RNA gene, partial sequence | Lactococcus lactis | 2682 | 2682 | 99% | 0 | 100 | 1523 | [JX267125.1](https://www.ncbi.nlm.nih.gov/nucleotide/JX267125.1?report=genbank&log$=nucltop&blast_rank=47&RID=FYETSPVH016) |
| Lactococcus lactis subsp. lactis IO-1 DNA, complete genome | Lactococcus lactis subsp. lactis IO-1 | 2682 | 16094 | 99% | 0 | 100 | 2421471 | [AP012281.1](https://www.ncbi.nlm.nih.gov/nucleotide/AP012281.1?report=genbank&log$=nucltop&blast_rank=48&RID=FYETSPVH016) |
| Lactococcus lactis strain LLKS2 16S ribosomal RNA gene, partial sequence | Lactococcus lactis | 2682 | 2682 | 99% | 0 | 100 | 1508 | [MK977610.1](https://www.ncbi.nlm.nih.gov/nucleotide/MK977610.1?report=genbank&log$=nucltop&blast_rank=49&RID=FYETSPVH016) |
| Lactococcus lactis strain R-707-1 chromosome, complete genome | Lactococcus lactis | 2682 | 16094 | 99% | 0 | 100 | 2457103 | [CP102522.1](https://www.ncbi.nlm.nih.gov/nucleotide/CP102522.1?report=genbank&log$=nucltop&blast_rank=50&RID=FYETSPVH016) |
| Lactococcus lactis strain SCB469 chromosome, complete genome | Lactococcus lactis | 2682 | 16037 | 99% | 0 | 100 | 2457093 | [CP094474.1](https://www.ncbi.nlm.nih.gov/nucleotide/CP094474.1?report=genbank&log$=nucltop&blast_rank=51&RID=FYETSPVH016) |
| Lactococcus lactis strain K_LL005 chromosome, complete genome | Lactococcus lactis | 2682 | 16094 | 99% | 0 | 100 | 2375093 | [CP060580.1](https://www.ncbi.nlm.nih.gov/nucleotide/CP060580.1?report=genbank&log$=nucltop&blast_rank=52&RID=FYETSPVH016) |
| Lactococcus lactis subsp. lactis strain 14B4 chromosome, complete genome | Lactococcus lactis subsp. lactis | 2682 | 16094 | 99% | 0 | 100 | 2579381 | [CP028160.1](https://www.ncbi.nlm.nih.gov/nucleotide/CP028160.1?report=genbank&log$=nucltop&blast_rank=53&RID=FYETSPVH016) |
| Lactococcus lactis subsp. lactis strain UC109 chromosome, complete genome | Lactococcus lactis subsp. lactis | 2682 | 16094 | 99% | 0 | 100 | 2392933 | [CP091107.1](https://www.ncbi.nlm.nih.gov/nucleotide/CP091107.1?report=genbank&log$=nucltop&blast_rank=54&RID=FYETSPVH016) |
| Lactococcus lactis strain ABRIINW.N6 16S ribosomal RNA gene, partial sequence | Lactococcus lactis | 2682 | 2682 | 99% | 0 | 100 | 1474 | [MK367684.1](https://www.ncbi.nlm.nih.gov/nucleotide/MK367684.1?report=genbank&log$=nucltop&blast_rank=55&RID=FYETSPVH016) |
| Lactococcus lactis strain 17M1 chromosome, complete genome | Lactococcus lactis | 2682 | 16087 | 99% | 0 | 100 | 2324726 | [CP092748.1](https://www.ncbi.nlm.nih.gov/nucleotide/CP092748.1?report=genbank&log$=nucltop&blast_rank=56&RID=FYETSPVH016) |
| Lactococcus lactis strain 2B-5 chromosome, complete genome | Lactococcus lactis | 2682 | 16094 | 99% | 0 | 100 | 2623329 | [CP157498.1](https://www.ncbi.nlm.nih.gov/nucleotide/CP157498.1?report=genbank&log$=nucltop&blast_rank=57&RID=FYETSPVH016) |
| Lactococcus lactis subsp. lactis strain G50 chromosome, complete genome | Lactococcus lactis subsp. lactis | 2682 | 16094 | 99% | 0 | 100 | 2346663 | [CP025500.1](https://www.ncbi.nlm.nih.gov/nucleotide/CP025500.1?report=genbank&log$=nucltop&blast_rank=58&RID=FYETSPVH016) |
| Lactococcus lactis strain TSB6 16S ribosomal RNA gene, partial sequence | Lactococcus lactis | 2682 | 2682 | 99% | 0 | 100 | 1511 | [JX291541.1](https://www.ncbi.nlm.nih.gov/nucleotide/JX291541.1?report=genbank&log$=nucltop&blast_rank=59&RID=FYETSPVH016) |
| Lactococcus lactis subsp. lactis strain UL8, complete genome | Lactococcus lactis subsp. lactis | 2682 | 16094 | 99% | 0 | 100 | 2422150 | [CP015908.1](https://www.ncbi.nlm.nih.gov/nucleotide/CP015908.1?report=genbank&log$=nucltop&blast_rank=60&RID=FYETSPVH016) |
| Lactococcus lactis strain CICC6036 16S ribosomal RNA gene, partial sequence | Lactococcus lactis | 2680 | 2680 | 99% | 0 | 100 | 1478 | [DQ212978.1](https://www.ncbi.nlm.nih.gov/nucleotide/DQ212978.1?report=genbank&log$=nucltop&blast_rank=61&RID=FYETSPVH016) |
| Lactococcus lactis subsp. lactis strain 1A2 16S ribosomal RNA gene, partial sequence | Lactococcus lactis subsp. lactis | 2680 | 2680 | 99% | 0 | 100 | 1505 | [EU337108.1](https://www.ncbi.nlm.nih.gov/nucleotide/EU337108.1?report=genbank&log$=nucltop&blast_rank=62&RID=FYETSPVH016) |
| Lactococcus lactis strain CICC6025 16S ribosomal RNA gene, partial sequence | Lactococcus lactis | 2680 | 2680 | 99% | 0 | 100 | 1482 | [DQ212981.1](https://www.ncbi.nlm.nih.gov/nucleotide/DQ212981.1?report=genbank&log$=nucltop&blast_rank=63&RID=FYETSPVH016) |
| Lactococcus lactis subsp. lactis strain A34 16S ribosomal RNA gene, partial sequence | Lactococcus lactis subsp. lactis | 2680 | 2680 | 99% | 0 | 100 | 1488 | [GU735481.2](https://www.ncbi.nlm.nih.gov/nucleotide/GU735481.2?report=genbank&log$=nucltop&blast_rank=64&RID=FYETSPVH016) |
| Lactococcus lactis strain CICC6030 16S ribosomal RNA gene, partial sequence | Lactococcus lactis | 2680 | 2680 | 99% | 0 | 100 | 1487 | [DQ212980.1](https://www.ncbi.nlm.nih.gov/nucleotide/DQ212980.1?report=genbank&log$=nucltop&blast_rank=65&RID=FYETSPVH016) |
| Lactococcus sp. 006.30 16S ribosomal RNA gene, partial sequence | Lactococcus sp. 006.30 | 2678 | 2678 | 99% | 0 | 100 | 1482 | [KU639600.1](https://www.ncbi.nlm.nih.gov/nucleotide/KU639600.1?report=genbank&log$=nucltop&blast_rank=66&RID=FYETSPVH016) |
| Lactococcus lactis strain FDAARGOS_865 chromosome, complete genome | Lactococcus lactis | 2676 | 16055 | 99% | 0 | 99.93 | 2518866 | [CP065737.1](https://www.ncbi.nlm.nih.gov/nucleotide/CP065737.1?report=genbank&log$=nucltop&blast_rank=67&RID=FYETSPVH016) |
| Lactococcus lactis strain VHProbi V60 chromosome, complete genome | Lactococcus lactis | 2676 | 16050 | 99% | 0 | 99.93 | 2416269 | [CP110849.1](https://www.ncbi.nlm.nih.gov/nucleotide/CP110849.1?report=genbank&log$=nucltop&blast_rank=68&RID=FYETSPVH016) |
| Lactococcus lactis subsp. lactis bv. diacetylactis strain S50 chromosome, complete genome | Lactococcus lactis subsp. lactis bv. diacetylactis | 2676 | 16061 | 99% | 0 | 99.93 | 2461759 | [CP061322.1](https://www.ncbi.nlm.nih.gov/nucleotide/CP061322.1?report=genbank&log$=nucltop&blast_rank=69&RID=FYETSPVH016) |
| Lactococcus lactis strain AK-40 16S ribosomal RNA gene, partial sequence | Lactococcus lactis | 2676 | 2676 | 99% | 0 | 99.93 | 1464 | [MZ350166.1](https://www.ncbi.nlm.nih.gov/nucleotide/MZ350166.1?report=genbank&log$=nucltop&blast_rank=70&RID=FYETSPVH016) |
| Lactococcus lactis subsp. lactis strain S1-G-2 16S ribosomal RNA gene, partial sequence | Lactococcus lactis subsp. lactis | 2676 | 2676 | 99% | 0 | 99.93 | 1468 | [OK326335.1](https://www.ncbi.nlm.nih.gov/nucleotide/OK326335.1?report=genbank&log$=nucltop&blast_rank=71&RID=FYETSPVH016) |
| Lactococcus lactis subsp. lactis bv. diacetylactis strain SD96 chromosome, complete genome | Lactococcus lactis subsp. lactis bv. diacetylactis | 2676 | 16061 | 99% | 0 | 99.93 | 2416272 | [CP043523.1](https://www.ncbi.nlm.nih.gov/nucleotide/CP043523.1?report=genbank&log$=nucltop&blast_rank=72&RID=FYETSPVH016) |
| Lactococcus lactis strain NCK401 chromosome, complete genome | Lactococcus lactis | 2676 | 16061 | 99% | 0 | 99.93 | 2551975 | [CP137623.1](https://www.ncbi.nlm.nih.gov/nucleotide/CP137623.1?report=genbank&log$=nucltop&blast_rank=73&RID=FYETSPVH016) |
| Lactococcus lactis strain HBUR51203 16S ribosomal RNA gene, partial sequence | Lactococcus lactis | 2676 | 2676 | 99% | 0 | 99.93 | 1473 | [OR502257.1](https://www.ncbi.nlm.nih.gov/nucleotide/OR502257.1?report=genbank&log$=nucltop&blast_rank=74&RID=FYETSPVH016) |
| Lactococcus sp. strain C2-G-5 16S ribosomal RNA gene, partial sequence | Lactococcus sp. | 2676 | 2676 | 99% | 0 | 99.93 | 1497 | [OK326118.1](https://www.ncbi.nlm.nih.gov/nucleotide/OK326118.1?report=genbank&log$=nucltop&blast_rank=75&RID=FYETSPVH016) |
| Lactococcus lactis subsp. lactis strain C20 chromosome, complete genome | Lactococcus lactis subsp. lactis | 2676 | 16061 | 99% | 0 | 99.93 | 2416264 | [CP142589.1](https://www.ncbi.nlm.nih.gov/nucleotide/CP142589.1?report=genbank&log$=nucltop&blast_rank=76&RID=FYETSPVH016) |
| Lactococcus lactis strain LJL7m20 chromosome, complete genome | Lactococcus lactis | 2676 | 16055 | 99% | 0 | 99.93 | 2433390 | [CP104388.1](https://www.ncbi.nlm.nih.gov/nucleotide/CP104388.1?report=genbank&log$=nucltop&blast_rank=77&RID=FYETSPVH016) |
| Lactococcus lactis subsp. lactis strain IMAU11823 chromosome, complete genome | Lactococcus lactis subsp. lactis | 2676 | 16061 | 99% | 0 | 99.93 | 2458520 | [CP041759.1](https://www.ncbi.nlm.nih.gov/nucleotide/CP041759.1?report=genbank&log$=nucltop&blast_rank=78&RID=FYETSPVH016) |
| Lactococcus lactis strain AH1 chromosome, complete genome | Lactococcus lactis | 2676 | 16061 | 99% | 0 | 99.93 | 2421519 | [CP093413.1](https://www.ncbi.nlm.nih.gov/nucleotide/CP093413.1?report=genbank&log$=nucltop&blast_rank=79&RID=FYETSPVH016) |
| Lactococcus lactis strain B26 chromosome, complete genome | Lactococcus lactis | 2676 | 16061 | 99% | 0 | 99.93 | 2370090 | [CP138309.1](https://www.ncbi.nlm.nih.gov/nucleotide/CP138309.1?report=genbank&log$=nucltop&blast_rank=80&RID=FYETSPVH016) |
| Lactococcus lactis strain MA5 chromosome, complete genome | Lactococcus lactis | 2676 | 16061 | 99% | 0 | 99.93 | 2322800 | [CP121539.1](https://www.ncbi.nlm.nih.gov/nucleotide/CP121539.1?report=genbank&log$=nucltop&blast_rank=81&RID=FYETSPVH016) |
| Lactococcus lactis subsp. lactis gene for 16S ribosomal RNA, partial sequence, strain: JCM 20128 | Lactococcus lactis subsp. lactis | 2676 | 2676 | 99% | 0 | 99.93 | 1498 | [LC311730.1](https://www.ncbi.nlm.nih.gov/nucleotide/LC311730.1?report=genbank&log$=nucltop&blast_rank=82&RID=FYETSPVH016) |
| Lactococcus lactis strain LA0312 chromosome | Lactococcus lactis | 2676 | 16061 | 99% | 0 | 99.93 | 2564033 | [CP134164.1](https://www.ncbi.nlm.nih.gov/nucleotide/CP134164.1?report=genbank&log$=nucltop&blast_rank=83&RID=FYETSPVH016) |
| Lactococcus lactis subsp. lactis strain N1-G-1 16S ribosomal RNA gene, partial sequence | Lactococcus lactis subsp. lactis | 2676 | 2676 | 99% | 0 | 99.93 | 1496 | [OK326226.1](https://www.ncbi.nlm.nih.gov/nucleotide/OK326226.1?report=genbank&log$=nucltop&blast_rank=84&RID=FYETSPVH016) |
| Lactococcus lactis subsp. lactis bv. diacetylactis strain FM03, complete genome | Lactococcus lactis subsp. lactis bv. diacetylactis | 2676 | 16061 | 99% | 0 | 99.93 | 2428349 | [CP020604.1](https://www.ncbi.nlm.nih.gov/nucleotide/CP020604.1?report=genbank&log$=nucltop&blast_rank=85&RID=FYETSPVH016) |
| Lactococcus lactis strain CJNU 3001 16S ribosomal RNA gene, partial sequence | Lactococcus lactis | 2676 | 2676 | 99% | 0 | 99.93 | 1546 | [MN749817.1](https://www.ncbi.nlm.nih.gov/nucleotide/MN749817.1?report=genbank&log$=nucltop&blast_rank=86&RID=FYETSPVH016) |
| Lactococcus lactis strain TBZ1 16S ribosomal RNA gene, partial sequence | Lactococcus lactis | 2676 | 2676 | 99% | 0 | 99.93 | 1492 | [KU291415.1](https://www.ncbi.nlm.nih.gov/nucleotide/KU291415.1?report=genbank&log$=nucltop&blast_rank=87&RID=FYETSPVH016) |
| Lactococcus lactis subsp. lactis strain CCMMB1030 16S ribosomal RNA gene, partial sequence | Lactococcus lactis subsp. lactis | 2676 | 2676 | 99% | 0 | 99.93 | 1510 | [KF879107.1](https://www.ncbi.nlm.nih.gov/nucleotide/KF879107.1?report=genbank&log$=nucltop&blast_rank=88&RID=FYETSPVH016) |
| Uncultured Staphylococcus sp. clone HEM319 16S ribosomal RNA gene, partial sequence; mitochondrial | uncultured Staphylococcus sp. | 2676 | 2676 | 99% | 0 | 99.93 | 1486 | [MF148183.1](https://www.ncbi.nlm.nih.gov/nucleotide/MF148183.1?report=genbank&log$=nucltop&blast_rank=89&RID=FYETSPVH016) |
| Lactococcus lactis subsp. lactis strain CCMMB1033 16S ribosomal RNA gene, partial sequence | Lactococcus lactis subsp. lactis | 2676 | 2676 | 99% | 0 | 99.93 | 1510 | [KF879109.1](https://www.ncbi.nlm.nih.gov/nucleotide/KF879109.1?report=genbank&log$=nucltop&blast_rank=90&RID=FYETSPVH016) |
| Lactococcus lactis strain FDAARGOS_866 chromosome, complete genome | Lactococcus lactis | 2676 | 16061 | 99% | 0 | 99.93 | 2364555 | [CP065735.1](https://www.ncbi.nlm.nih.gov/nucleotide/CP065735.1?report=genbank&log$=nucltop&blast_rank=91&RID=FYETSPVH016) |
| Lactococcus lactis strain N8 chromosome, complete genome | Lactococcus lactis | 2676 | 16061 | 99% | 0 | 99.93 | 2421567 | [CP059049.1](https://www.ncbi.nlm.nih.gov/nucleotide/CP059049.1?report=genbank&log$=nucltop&blast_rank=92&RID=FYETSPVH016) |
| Lactococcus lactis strain TCAN05 16S ribosomal RNA gene, partial sequence | Lactococcus lactis | 2676 | 2676 | 99% | 0 | 99.93 | 1523 | [OM992236.1](https://www.ncbi.nlm.nih.gov/nucleotide/OM992236.1?report=genbank&log$=nucltop&blast_rank=93&RID=FYETSPVH016) |
| Lactococcus lactis subsp. lactis strain 229 chromosome, complete genome | Lactococcus lactis subsp. lactis | 2676 | 16050 | 99% | 0 | 99.93 | 2514313 | [CP090823.2](https://www.ncbi.nlm.nih.gov/nucleotide/CP090823.2?report=genbank&log$=nucltop&blast_rank=94&RID=FYETSPVH016) |
| Lactococcus lactis subsp. lactis strain IHB B 15952 16S ribosomal RNA gene, partial sequence | Lactococcus lactis subsp. lactis | 2676 | 2676 | 99% | 0 | 99.93 | 1519 | [KM817251.1](https://www.ncbi.nlm.nih.gov/nucleotide/KM817251.1?report=genbank&log$=nucltop&blast_rank=95&RID=FYETSPVH016) |
| Lactococcus lactis subsp. lactis bv. diacetylactis strain Ge001 chromosome, complete genome | Lactococcus lactis subsp. lactis bv. diacetylactis | 2676 | 16061 | 99% | 0 | 99.93 | 2409036 | [CP069378.1](https://www.ncbi.nlm.nih.gov/nucleotide/CP069378.1?report=genbank&log$=nucltop&blast_rank=96&RID=FYETSPVH016) |
| Lactococcus lactis subsp. lactis gene for 16S ribosomal RNA, partial sequence, strain: JCM 7638 | Lactococcus lactis subsp. lactis | 2676 | 2676 | 99% | 0 | 100 | 1486 | [LC096207.1](https://www.ncbi.nlm.nih.gov/nucleotide/LC096207.1?report=genbank&log$=nucltop&blast_rank=97&RID=FYETSPVH016) |
| Lactococcus lactis subsp. lactis strain 223 chromosome, complete genome | Lactococcus lactis subsp. lactis | 2676 | 16061 | 99% | 0 | 99.93 | 2474987 | [CP031926.2](https://www.ncbi.nlm.nih.gov/nucleotide/CP031926.2?report=genbank&log$=nucltop&blast_rank=98&RID=FYETSPVH016) |
| Lactococcus lactis subsp. lactis NCDO 2118, complete genome | Lactococcus lactis subsp. lactis NCDO 2118 | 2676 | 16061 | 99% | 0 | 99.93 | 2554693 | [CP009054.1](https://www.ncbi.nlm.nih.gov/nucleotide/CP009054.1?report=genbank&log$=nucltop&blast_rank=99&RID=FYETSPVH016) |
| Lactococcus lactis subsp. lactis strain G121 chromosome, complete genome | Lactococcus lactis subsp. lactis | 2676 | 16054 | 99% | 0 | 99.93 | 2573315 | [CP053671.2](https://www.ncbi.nlm.nih.gov/nucleotide/CP053671.2?report=genbank&log$=nucltop&blast_rank=100&RID=FYETSPVH016) |
