# Supplementary figures and images for: Intestinal Lactobacillus johnsonii protects against neuroangiostrongyliasis in BALB/c mice through modulation of immune response
Source: PLoS Negl Trop Dis. 2025 Apr 8;19(4):e0012977. doi: 10.1371/journal.pntd.0012977 (PMC11978024; doi:10.1371/journal.pntd.0012977)

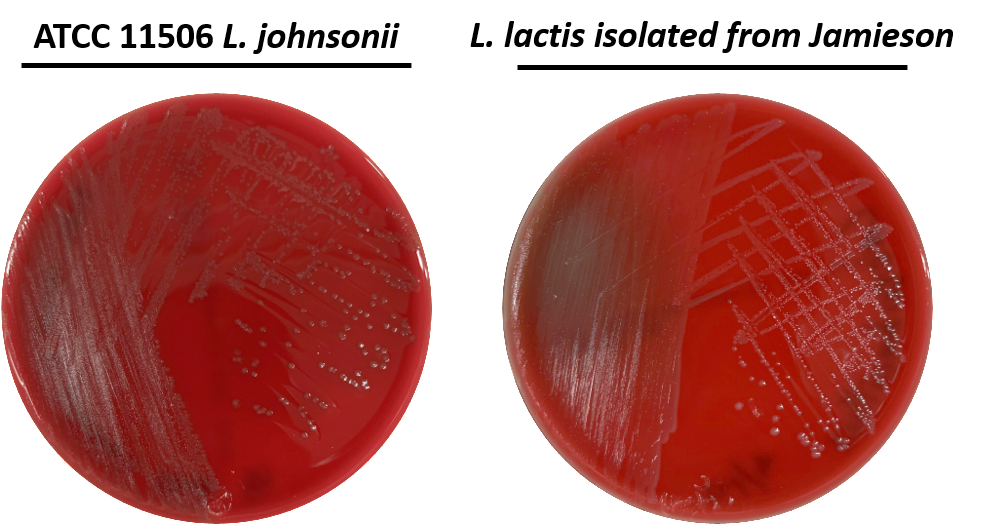

Supplement: S1 Fig — (TIF) [file pntd.0012977.s010.tif]

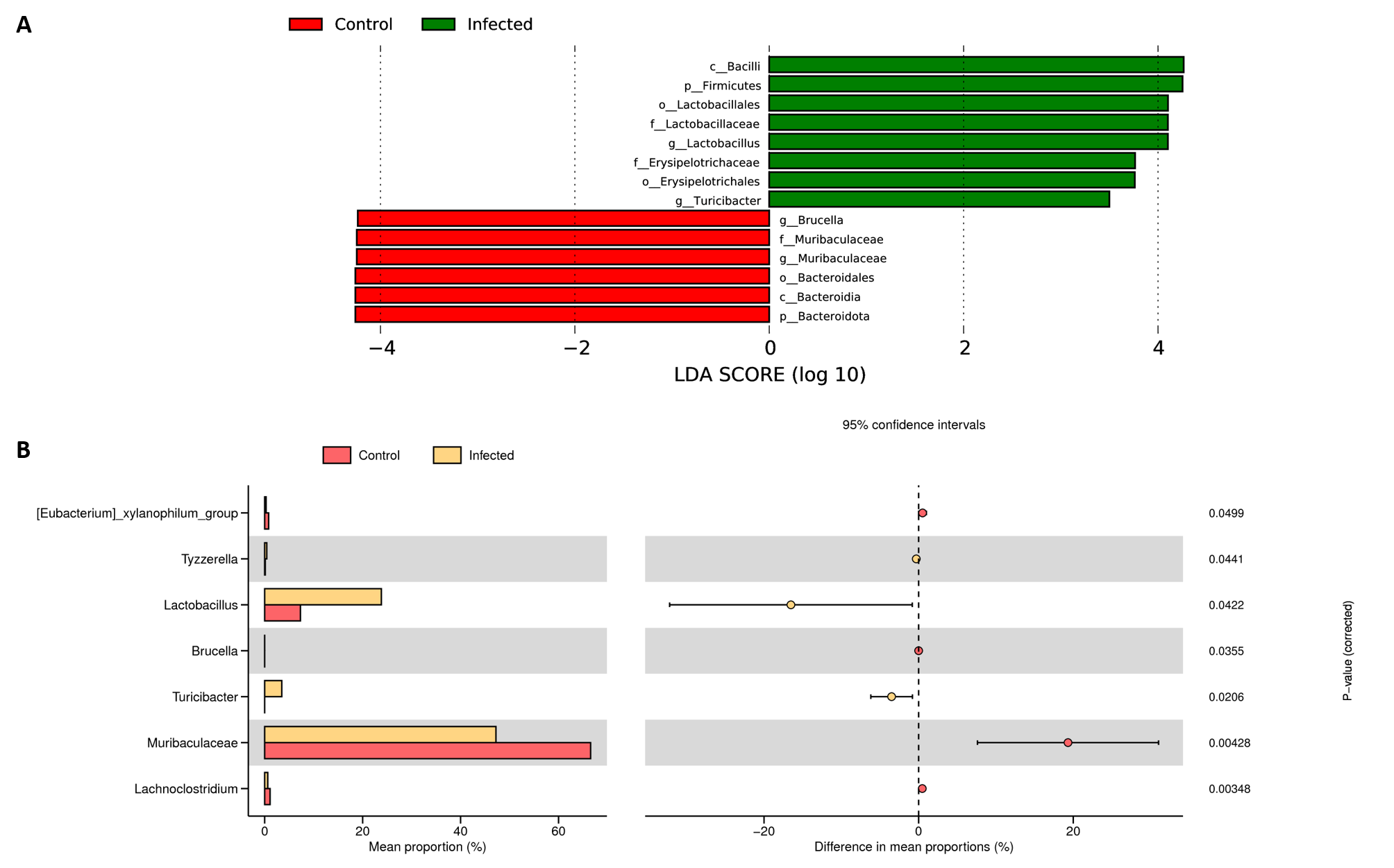

Supplement: S2 Fig — The horizontal coordinate shows the logarithmic LDA scores for each taxonomic unit. The longer lengths indicate the more significant differences in the classification. The default setting was LDA effect size > 3 and p-value < 0.05. (B) STAMP analysis at the genus levels showing significant differences in the microbiome abundance between uninfected and A. cantonensis-infected mice. The left figure shows the percentage of mean proportion of bacterial strains in the two groups; the right figure shows the percentage of difference of mean proportion of bacterial genera within the 95% confidence interval; p-values are shown on the right. n = 6 mice in both groups. Significance determined by Welch’s t-test, with p < 0.05 considered significant. (TIF) [file pntd.0012977.s011.tif]

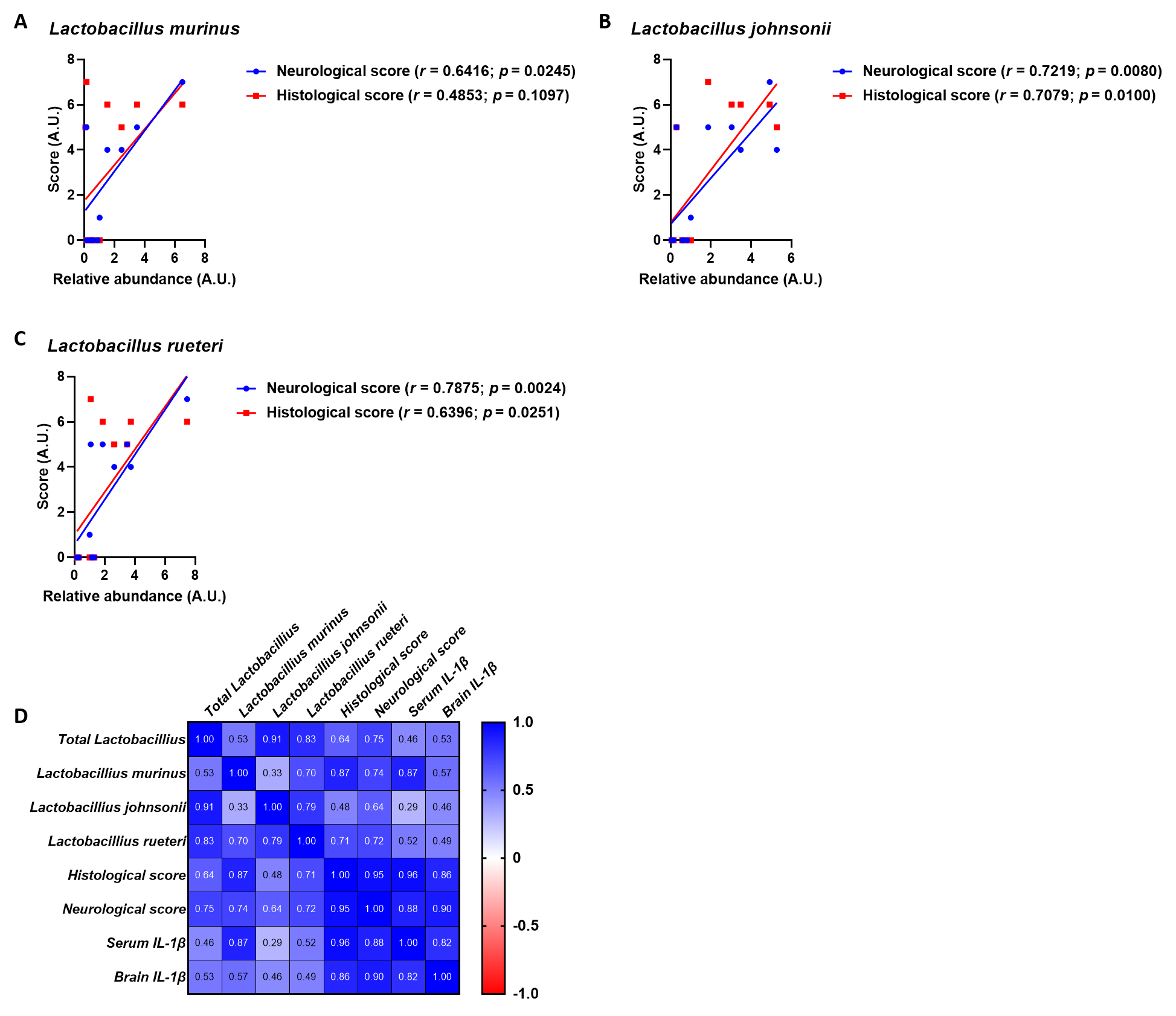

Supplement: S3 Fig — (D) Pearson’s correlation matrix between different variables. Pearson correlation coefficient values (r) are shaded with different color codes: positive correlations are from white to blue on the color scale, and negative correlations are from white to red. No negative correlation was seen between these variables. Bacterial abundance was qPCR data. A.U., arbitrary unit. n = 6 mice in the uninfected and infected group. (TIF) [file pntd.0012977.s012.tif]

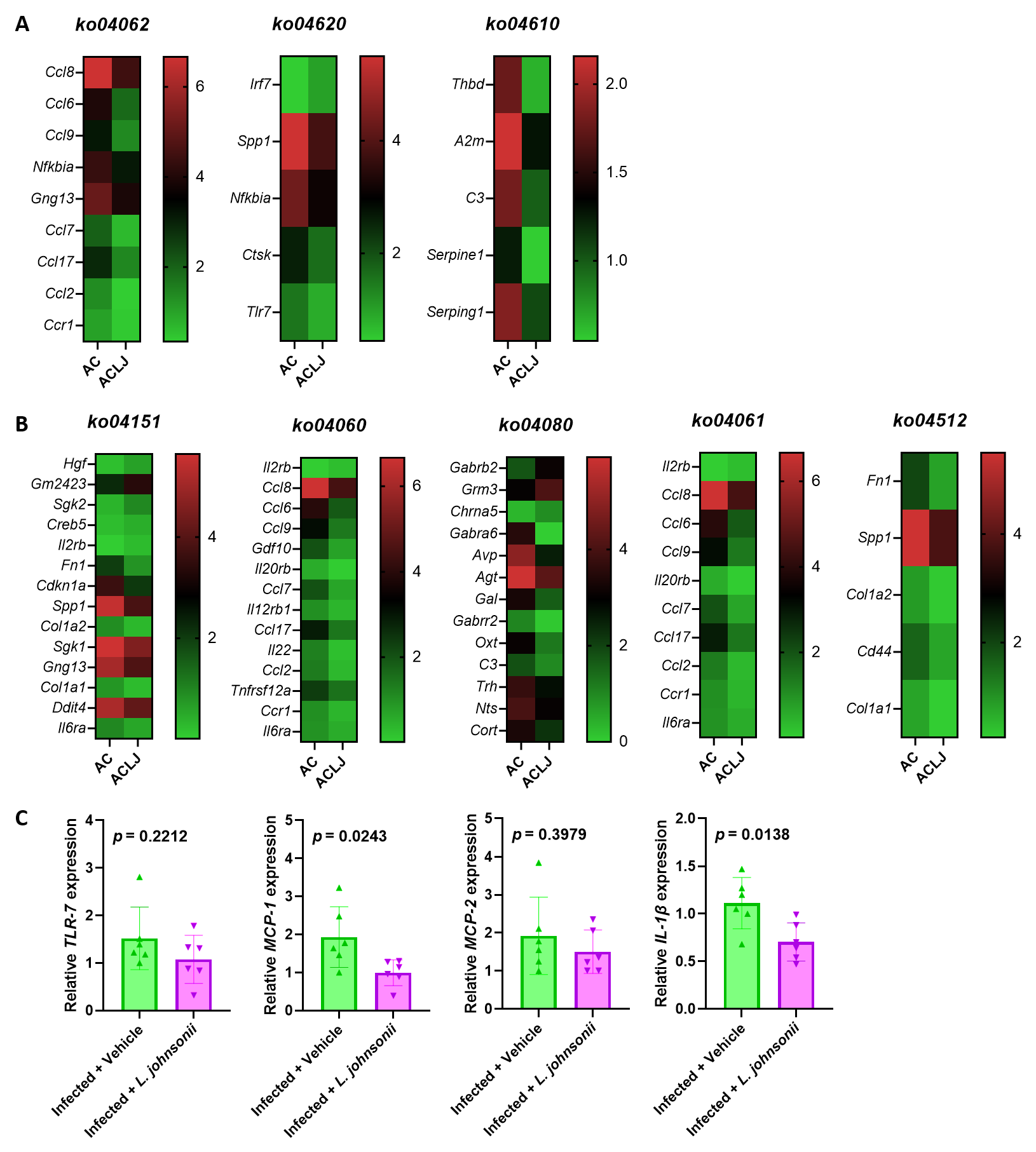

Supplement: S4 Fig — (A) Identified pathways within the organismal systems level including chemokine signaling pathway (ko04062), toll-like receptor signaling pathway (ko04620), and complement and coagulation cascades (ko04610). (B) Identified pathways within the environmental information processing level including PI3K-Akt signaling pathway (ko04151), cytokine-cytokine receptor interaction (ko04060), neuroactive ligand-receptor interaction (ko04080), viral protein interaction with cytokine and cytokine receptor (ko04061), and ECM-receptor interaction (ko04512). AC, brain from Angiostrongylus cantonensis-infected mice; ACLJ, brain from Lactobacillus johnsonii-treated, A. cantonensis-infected mice. Gene expression levels are shaded with different colors on a color scale from green (lower expression) to red (higher expression). (C) qPCR validation of selected genes. Data are presented as mean ± S.D. n = 6 mice in both groups. Significance determined by unpaired T-test, with p < 0.05 considered significant. (TIF) [file pntd.0012977.s013.tif]

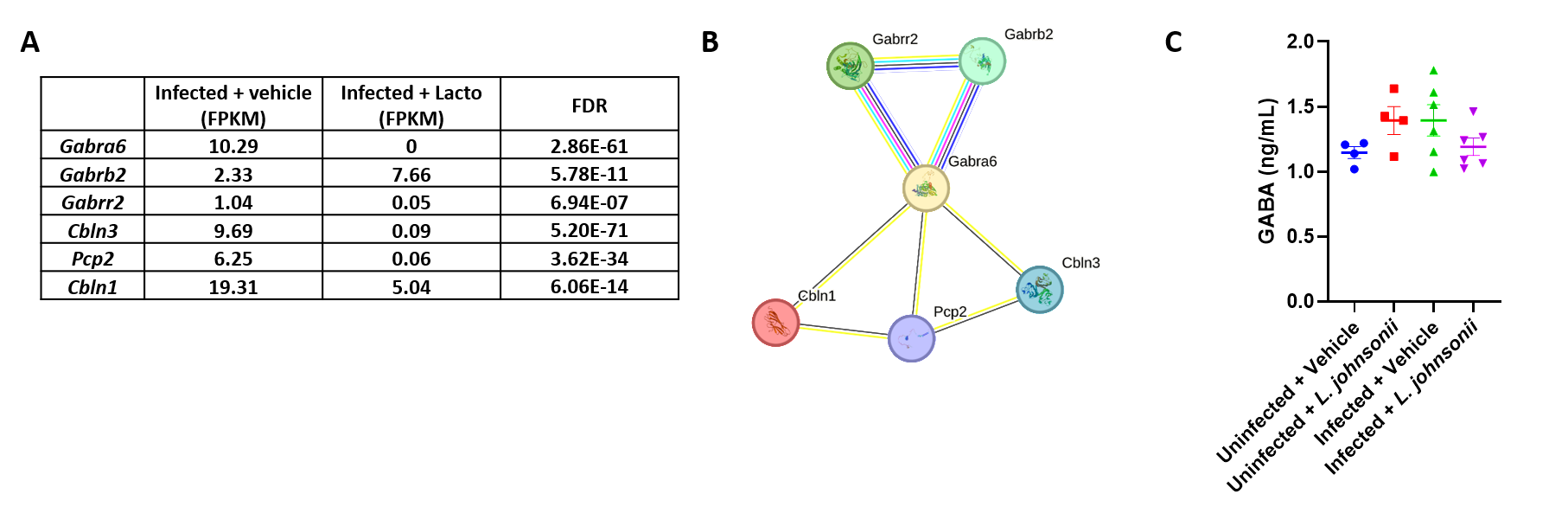

Supplement: S5 Fig — cantonensis-infected mice. (B) Network interaction map showing potential protein-protein interaction of the identified transcriptome in (A). The analysis was carried out with the publically available STRING database (https://string-db.org/; version 12.0). (C) Serum levels of GABA. n = 4 uninfected mice (with or without L. johnsonii inoculation) and n = 6 infected mice (with or without L. johnsonii inoculation). Data are presented as mean ± S.E.M. Significance determined by (A) false discovery rate (FDR) or (C) one-way ANOVA with Tukey’s honest significant difference test. FDR < 0.05 is considered significant. (TIF) [file pntd.0012977.s014.tif]

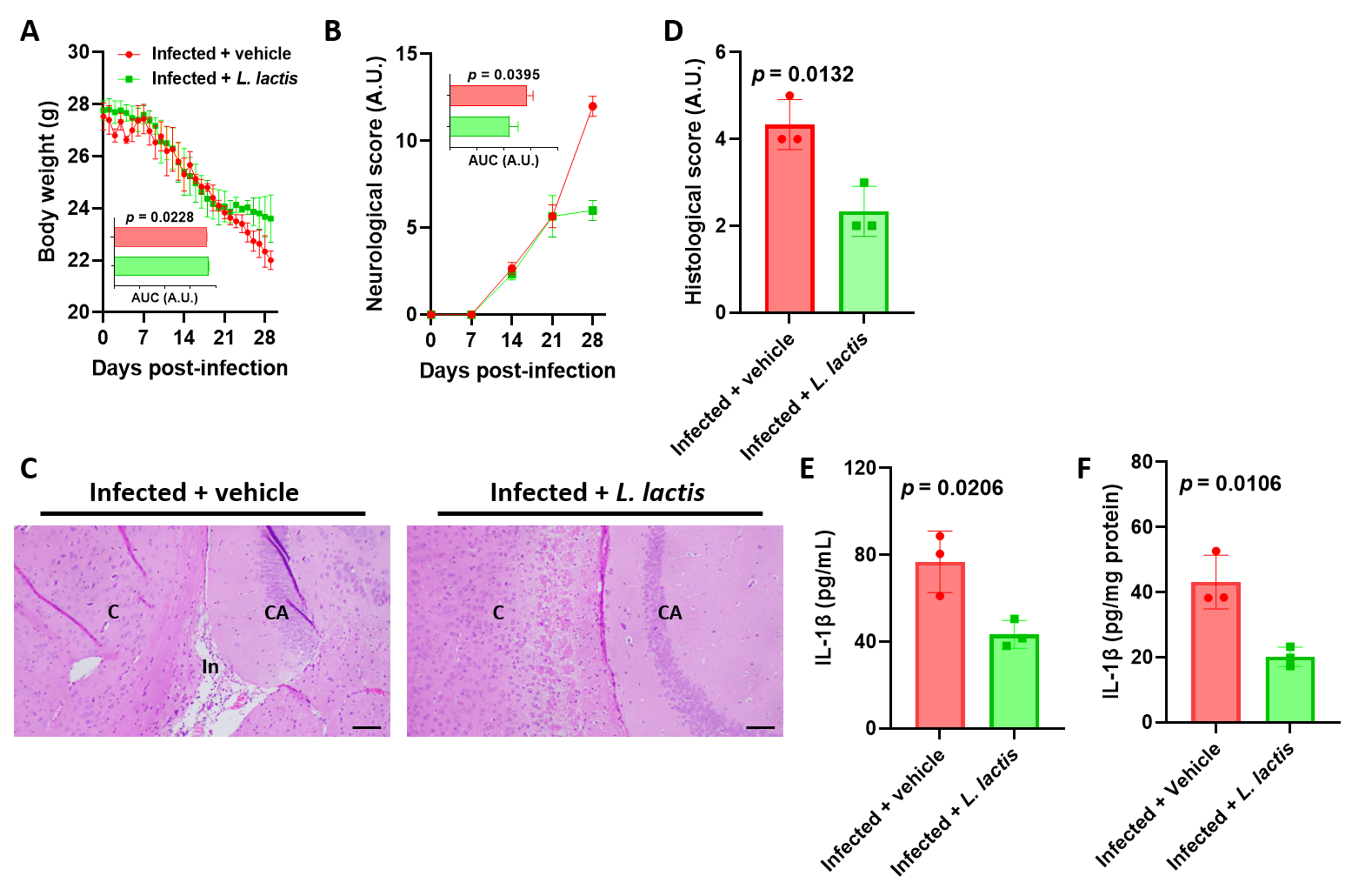

Supplement: S6 Fig — (A) The body weight of mice. (B) Neurological score of mice. (C) Representative H&E-stained section of the mouse brain. Scale bars correspond to 200 μm. (D) Histological score of the brain sections. (E-F) Serum (E) and brain (F) interleukin (IL)-1β levels. n = 3 mice in each group. Data in (A and B) are presented as mean ± S.E.M and (D-F) as mean ± S.D; area under curve (AUC) data are presented as mean ± S.D. A.U., arbitrary unit. Significance determined by unpaired T-test, with p < 0.05 considered significant. (TIF) [file pntd.0012977.s015.tif]
